# Development of an Interactive Software Tool for Designing Solvent Recovery Processes

**DOI:** 10.1021/acs.iecr.2c02920

**Published:** 2023-01-20

**Authors:** Jake P. Stengel, Austin L. Lehr, Emmanuel A. Aboagye, John D. Chea, Kirti M. Yenkie

**Affiliations:** Department of Chemical Engineering, Rowan University, 201 Mullica Hill Road, Glassboro, New Jersey08028, United States

## Abstract

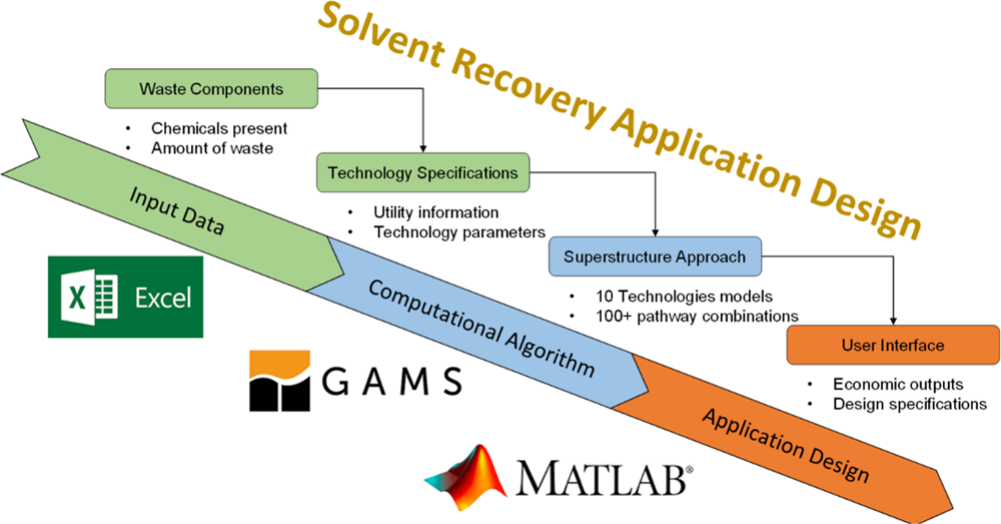

Solvents are used in chemical and pharmaceutical industries as a reaction medium, selective dissolution and extraction media, and dilution agents. Thus, a sizable amount of solvent waste is generated due to process inefficiencies. Most common ways of handling solvent waste are on-site, off-site disposal, and incineration, which have a considerable negative environmental impact. Solvent recovery is typically not used because of potential difficulties in achieving required purity guidelines, as well as additional infrastructure and investments that are needed. To this end, this problem must be studied carefully by involving aspects from capital needs, environmental benefits, and comparison with traditional disposal methods, while achieving the required purity. Thus, we have developed a user-friendly software tool that allows engineers to easily access solvent recovery options and predict an economical and environmentally favorable strategy, given a solvent-containing waste stream. This consists of a maximal process flow diagram that encompasses multiple stages of separations and technologies within those stages. This process flow diagram develops the superstructure that provides multiple technology pathway options for any solvent waste stream. Separation technologies are placed in different stages; depending on the component, they can separate in terms of their physical and chemical properties. A comprehensive chemical database is created to store all relevant chemical and physical properties. The pathway prediction is modeled as an economic optimization problem in General Algebraic Modeling Systems (GAMS). With GAMS code as the backend, a Graphical User Interface (GUI) is created in Matlab App Designer to provide a user-friendly tool to the chemical industry. This tool can act as a guidance system to assist professional engineers and provide an easy comparative estimate in the early stages of process design.

## 1 Introduction

**Figure 1:**
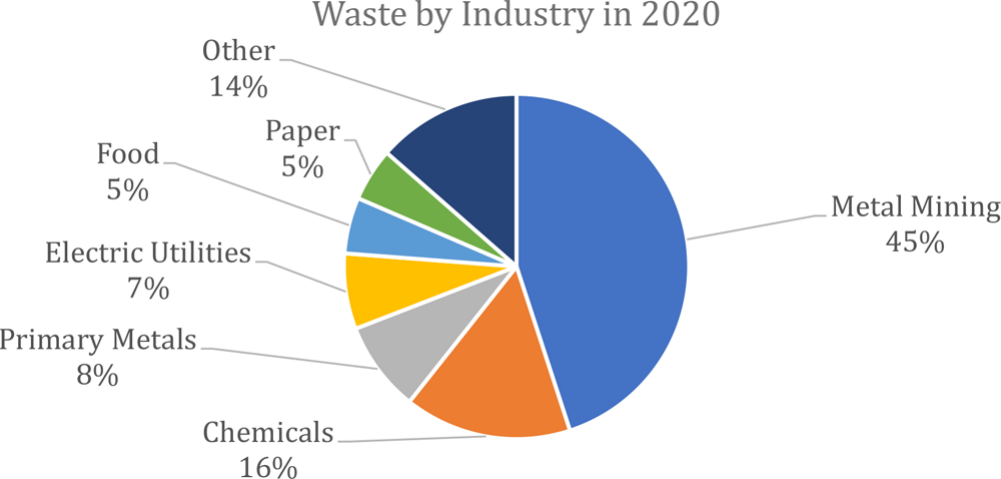
Waste breakdown of total releases by industry in 2020 (data provided by the USEPA in 2022^2^).

Industries and facilities generating hazardous wastes are under scrutiny and surveillance, because these wastes continue to increase without a sustainable disposal or recovery method. The United States currently manages ∼35 billion kilograms of hazardous materials annually. However, there are enough resources and land area to continue treatment and mitigate the effects of these wastes through the year 2044.^1^ Additionally, the ever-growing population demands an increase in goods, services, and medicines, leading to industries and facilities producing more products and, in turn, releasing more hazardous waste. To monitor the waste generated, the USEPA has been recording the releases of harmful chemicals since 1988. This information is stored in the Toxic Release Inventory (TRI).^2^ Using TRI, key industries releasing hazardous wastes can be identified, and more guidance and awareness resources can be created for these facilities. In turn, waste minimization efforts can be concentrated by switching to using greener chemicals and processes. Figure 1 displays the waste contribution from each major hazardous waste generator in 2020, which totals over 1.36 billion kilograms. The top three waste generating industries are the metal mining, chemicals, and primary metals industries.

**Figure 2:**
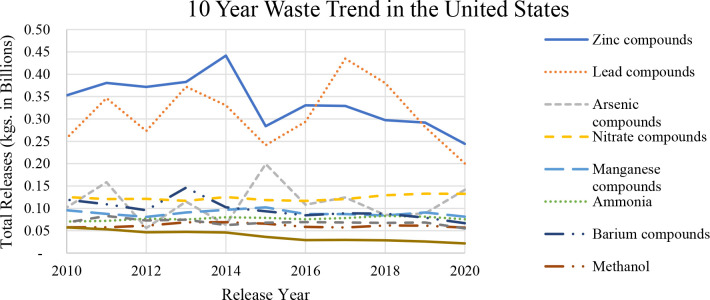
Trend of the top 10 waste chemicals released in the United States over a 10-year period (data provided by the USEPA in 2022^2^).

TRI was also used to examine the top ten chemical releases over 10 years, as shown in Figure 2. The top ten chemicals belong to two distinct categories: (i) metals or (ii) process byproducts. The process byproducts consist of chemicals used as a reaction medium within a manufacturing process. These substances are typically discarded post-consumer use, because they do not meet the purity specifications required for reusing them.^3−5^ As a result, considerable efforts have been made to decrease metal waste.

**Figure 3:**
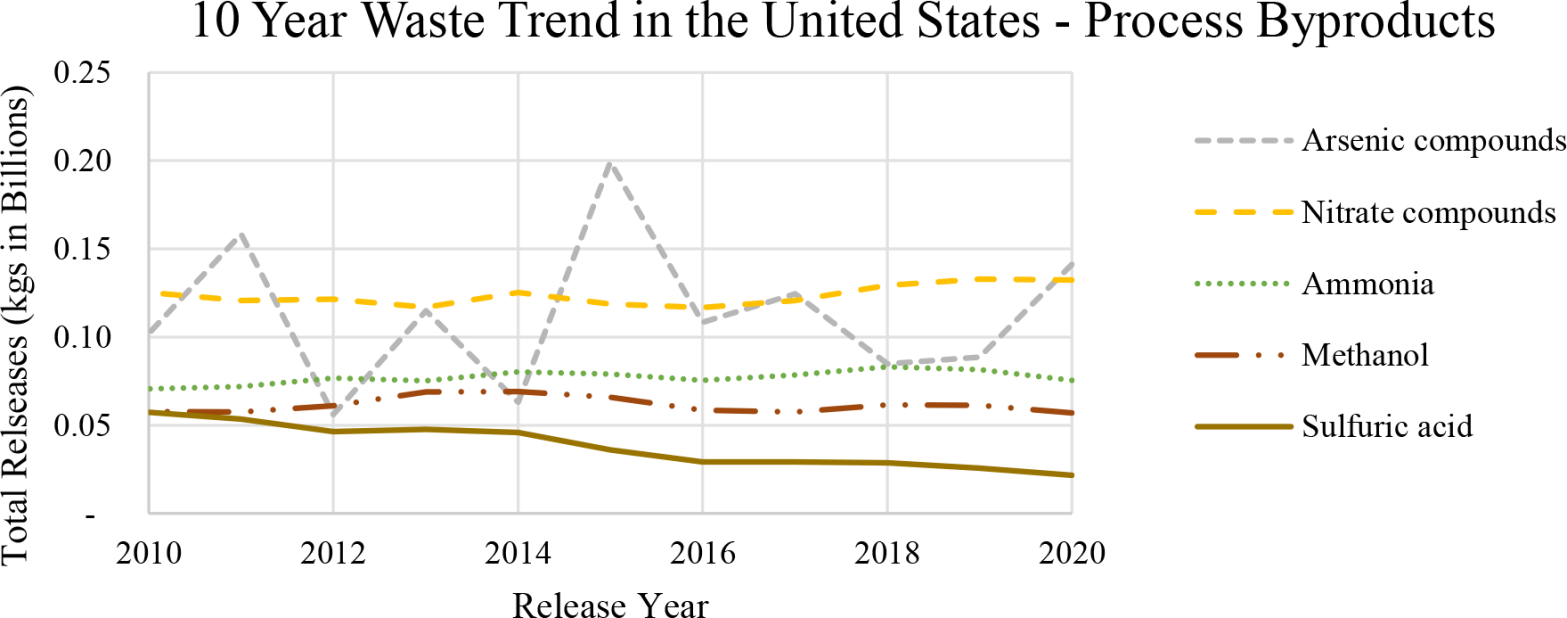
Trend of the top 5 waste process byproducts in the United States over a 10-year period (data provided by the USEPA in 2022^2^).

Additionally, some compounds have seen up to a 40% decline in waste generation from 2010 to 2020. However, for the process byproducts, this decline does not apply. Except for sulfuric acid, the majority of the chemicals in this category have not seen a decline with time, as observed in the metal waste category. Figure 3 shows the trend for all process byproduct materials from the top ten chemicals shown in Figure 2. In the case of the process byproducts, these hazardous materials generation has either remained steady or has increased over time, because the waste minimization methods cannot keep pace with the increase in demand for products to meet the growing population.

Solvents are a major group of chemicals contributing to the growing waste trend. Solvents are defined as a liquid that can either break the lattice structure of solid reactants, dissolve gaseous or liquid reactants, or exert considerable influence over reaction rates.^6^ The top three waste chemicals in the United States—ammonia, methanol, and sulfuric acid—contribute to nearly 15% of the total waste. Therefore, developing a new method for solvent waste handling can vastly decrease the overall hazardous materials production.

There are three widely used options for industries producing these wastes, with regard to solvent waste handling: on-site releases, off-site releases, and incineration. Each method does have some adverse effects on the environment and requires economic expenditures but is widely used since these options are easily accessible and have been prevalent for a long time. A production facility can release the waste directly into the land, air, or water for on-site releases. If injected into the land or a water body, it must be discharged below the lowest level of the available source of drinking water.^7,8^ In addition, the facility must abide by strict regulations about allowable chemical concentrations in those air emissions if dispersed into the air. For off-site release, the chemicals are often sold to a third party to dispose of the chemicals or use them at their facility in low-end processes. In industries with purity concerns, the solvents are often sold to other facilities where there are fewer purity regulations.^9^ The last method, incineration, is very effective with regard to disposing of most materials. Although a constant feed flow is needed for maximum efficiency, incineration can thermally decompose nearly 100% of volatile organic compounds and can recover thermal energy to be used for other equipment within the process.^10−12^ Although efficient, incineration is not environmentally friendly, because it can release harmful chemicals and pollutants into the atmosphere.^13,14^ Solvent recovery is a better alternative than the previous methods because it can improve the sustainability and greenness of chemical processes.^13,15,16^ In addition, solvent recovery poses economic benefits since fresh solvent will not need to be purchased in such high quantities anymore, because much of these recovered solvents can be reused. While many unique recovery methods have been researched, no standard solvent recovery method has been implemented.^9,17,18^

To this end, a solvent recovery tool can be a viable and quick solution to identifying solvent waste mitigation strategies to reduce the total amount of hazardous waste released into the environment. However, solvent recovery design is very complex, since it requires engineers to design a new system to implement in their facility. In addition, the engineer must collect, process, and analyze large amounts of information to get a feasible recovery option, which can be very time-consuming. Because of the fast-paced nature of design projects, many engineers do not have the time to analyze all available options within a limited time frame. We have previously developed a superstructure-based solvent recovery framework capable of suggesting an optimal recovery pathway with minimal cost and environmental impacts^15,16^ (refer to the Supporting Information for more details). The previous work can analyze multiple recovery options simultaneously within a short time frame. However, the approach is not user-friendly and requires the user to have prior knowledge of chemical engineering and coding experience. This work aims to provide a user-friendly approach to solvent recovery that can eliminate the need for high-level programming knowledge. By building this GUI around the solvent recovery framework, industries can start to explore and implement new solvent waste handling, recovery, and recycling methods.

## 2 Software Application Architecture

This section will outline the architecture and capabilities of this application in the following four subsections. Subsection 2.1 discusses the scope and limitations of the tool. Subsection 2.2 details the back-end of the application where the major modeling and optimization take place. Subsection 2.3 focuses on the software construction and how the coding platform interacts with the data storage and front-end interface. Subsection 2.4 discusses the software interface and the utility of each tab. It also demonstrates the inputs needed from the user and how to interpret the results from the tool.

### 2.1 Application Scope

This application was designed with the intention of giving engineers a quick, reliable tool to begin their design process. The beginning of any design project is dynamic as the project team is testing new ideas. The intention of this tool is to facilitate a quick estimation of possible technologies using a systematic approach rather than rely on past experience. This is all in an effort to expedite the design process by reducing the amount of guess-work and allow the engineers to apply their knowledge of their process to the solution presented from the tool.

### 2.2 Back-End Algorithm

**Figure 4:**
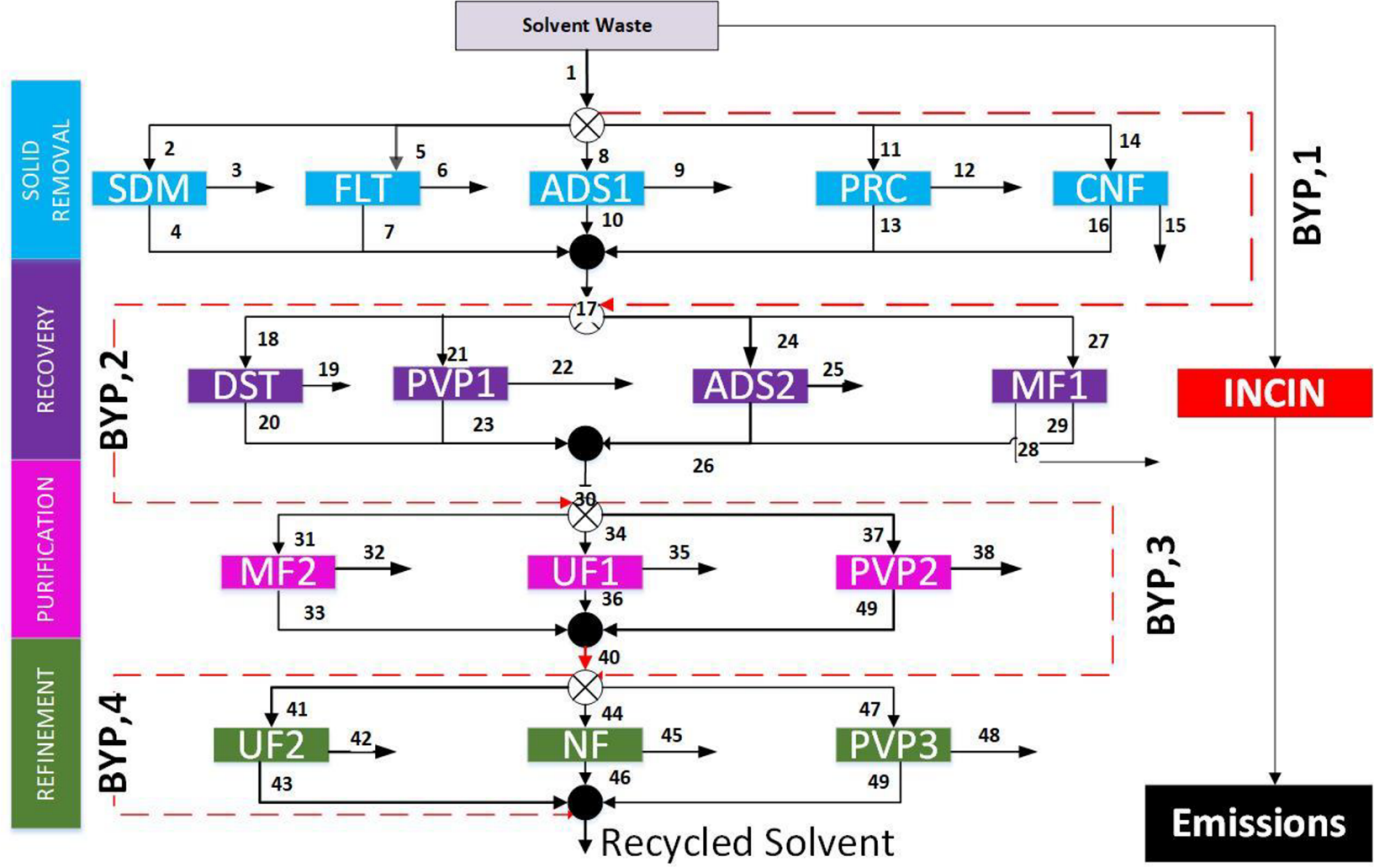
Solvent recovery superstructure. [Legend: SDM, sedimentation; FLT, filtration; ADS, adsorption; PRC, precipitation; CNF, centrifugation; DST, distillation; PVP, pervaporation; MF, microfiltration; UF, ultrafiltration; NF, nanofiltration; and BYP, bypass.]

The solvent recovery algorithm was developed in the General Algebraic Model Systems (GAMS) coding environment. Because of the complexity of the technology models,^19−21^ the program is modeled as a Mixed-Integer Nonlinear Program (MINLP)^22,23^ and solved using the Branch-and-Reduce Optimization Navigator (BARON).^24^ As highlighted in previous work, this algorithm is a superstructure approach to solvent recovery allowing the algorithm to examine all possible outcome.^15^ This approach allowed us to break the separation into four distinct stages: (i) Solid Removal,^25−27^ (ii) Recovery,^22,28−30^ (iii) Purification,^31^ and (iv) Refinement,^29,32^ as seen in Figure 4; more information is available in the Supporting Information.

The solid removal stage removed any solids from the waste stream, if present. The recovery stage did the major separations, which retained most of the solvent. The last two stages were there to reach any purity regulations the industry needed. If any stage was not needed, it could be bypassed completely, thus contributing no cost to the model. Using this four-stage superstructure approach, any liquid solvent waste stream could be analyzed, and economic results could be computed. From these economic results, justifications could be made on whether solvent recovery was a viable option for companies looking to implement the technique.

### 2.3 Software Construction

To use the algorithm to its full extent, in-depth knowledge of Excel, GAMS, and databases was needed. To alleviate the burden on the user, a GUI was developed to structure the inputs so the algorithm could read the information correctly. The foundation of this GUI was built using MATLAB app designer, because of the ease of communication between MATLAB, Excel, GAMS, and the database engine. MATLAB, Excel, and GAMS all have built-in functions that allow each coding environment to connect with one another, thus simplifying the program communications.

The MATLAB GUI was developed around the necessary inputs needed by the superstructure. These inputs were divided into two categories: waste specifications and the technology specifications (Table 1). The summarized breakdown included the waste specifications such as the compounds of interest, chemical properties, mass fraction, total mass flow, desired recovery/purity, chemical properties.^6,33,34^ These are the parameters that need be specified with each run to get an accurate estimate of the proposed solvent recovery method. Technology specifications (for utility information, consumable costs, maintenance times, standard cost/capacities, and operating parameters, please refer to the Supporting Information) were parameters that had default values, which the user could change.^35−38^ This would cut down on the number of inputs required by the user while giving the user freedom to modify the algorithm if they so choose. This also added the benefits of exposing engineers to different technologies they were not acquainted with and giving a reasonable basis for these unfamiliar technologies.

**Table 1:** Waste Specifications and Technology Specifications Variables and Parameters

waste specifications	technology specifications
compounds present	utility information
chemical properties	operating parameters
desired recovery/purity	consumables
total mass or mass flow	maintenance times
mass fraction for each compound	standard costs and capacities

**Figure 5:**
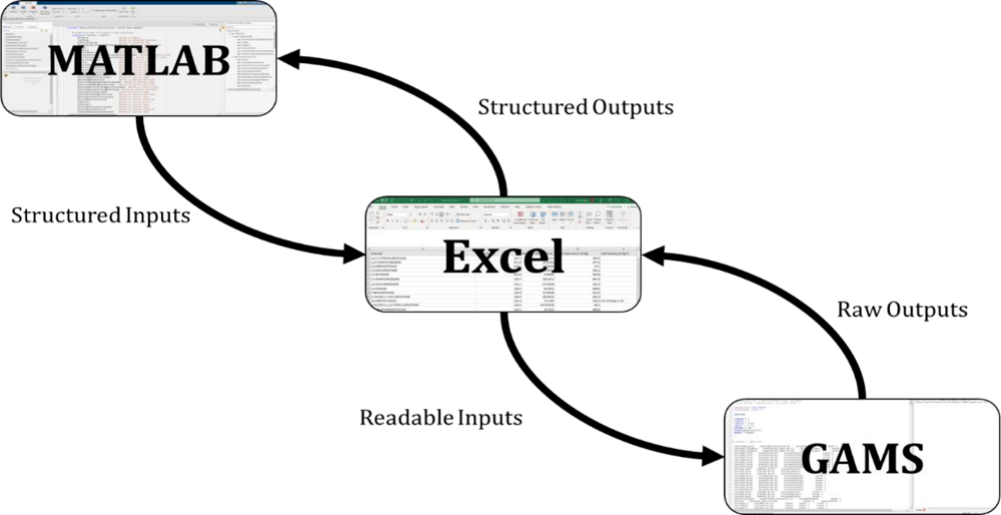
Communication network between independent coding environments and software.

After solidifying all the inputs, the team mapped out the optimal communication network needed by the coding environments. As Figure 5 depicts, MATLAB would take the waste specifications and technology specification inputs from the user and structure the inputs. Unfortunately, GAMS would not be able to read these structured inputs; therefore, an intermediate platform would need to be used. At the time of writing this paper, Excel was chosen, since Excel had existing functions that allowed for easy communication between the other software. While Excel is not a database management system, it serves as a suitable substitute until a database system can be implemented. Therefore, Excel only houses and organizes the data while MATLAB issues all the commands to run the GUI and the GAMS optimization program. With all the user inputs in Excel, built-in functions in GAMS could be used to read the data and transfer the information to the superstructure algorithm. Afterward, the algorithm is solved in GAMS, and results are generated. These results are sent back to Excel since they could not be directly interpreted by MATLAB. MATLAB was unable to read the complete results for the user, so this Excel sheet was used as an intermediary to record all the GAMS results so the GUI could report the optimization conclusions. Afterward, the user has all the information needed to judge whether a solvent waste handling and recovery method was viable or not for their process waste stream.

In addition to the GUI, a chemical database was developed to simplify the experience for the user. This database houses all the required physical and chemical properties by the computational algorithm and was constructed in Excel. The foundation of the database started with all harmful chemicals in the United States from the TRI database.^2^ Afterward, the Design Institute for Physical Properties (DIPPR) was used to acquire physical and chemical properties for each chemical obtained from TRI.^33^ Properties that were independent of temperature change, such as molecular weight, boiling point, and melting point, were recorded, if available. If a property was dependent on the temperature, the relation was recorded at room temperature. Later, additional chemicals and compounds were added to create a well-rounded library of chemicals. This allowed a user to search for chemicals from the database rather than inputting in all the necessary information to use the tool, simplifying the whole process.

### 2.4 Software Interface

**Figure 6:**
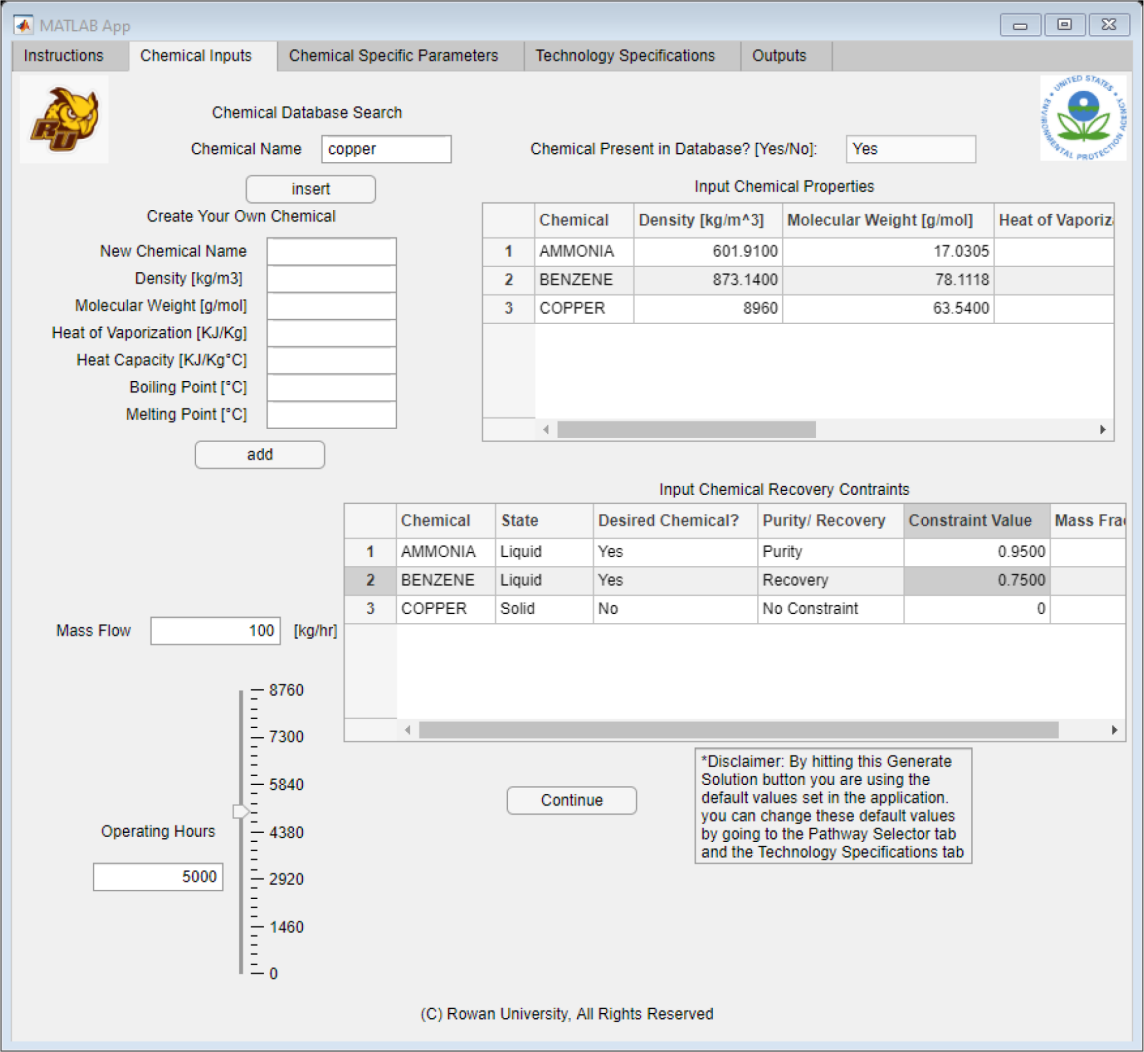
Application interface built using MATLAB App Designer, showing the “Chemical Inputs” tab.

The application was divided into four main separate tabs: Chemical Inputs, Chemical Specific Parameters, Technology Specifications, and Outputs. For the purpose of this example, a theoretical waste stream of ammonia, benzene, and copper was used to show the functionality of the application. The first tab was the “Chemical Inputs” tab, which allowed the user to input all the waste specifications. As Figure 6 shows, this table allowed the user to input all components present in the waste stream and define what components they would like to recover. The user could look up the different chemicals in the database by typing the chemical name in the chemical “database search field”. If present, a “yes” would populate in the text box next to the entry field. If the chemical was not in the database, the field would populate with “no” and the user would need to fill in the corresponding data on the lefthand side of the table under “Create Your Own Chemical”. Once all the fields have been entered, the user can click the “add” button to use the custom chemical in the algorithm. After the chemicals were imported, the user then would move onto the bottom table, labeled “Input Chemical Recovery Constraints”. This table allowed for the user to specify the state, if the chemical was desired, either a purity or recovery constraint, and the mass fraction. After the information was selected for each chemical, the user would need to enter a constraint value between 0 and 1 and specify the mass fraction between 0 and 1. After those constraints were filled out, the user would only need to input estimated operating hours and the continuous flow rate. Later, the user could click the “Continue” button to progress to the other tabs in the application.

**Figure 7:**
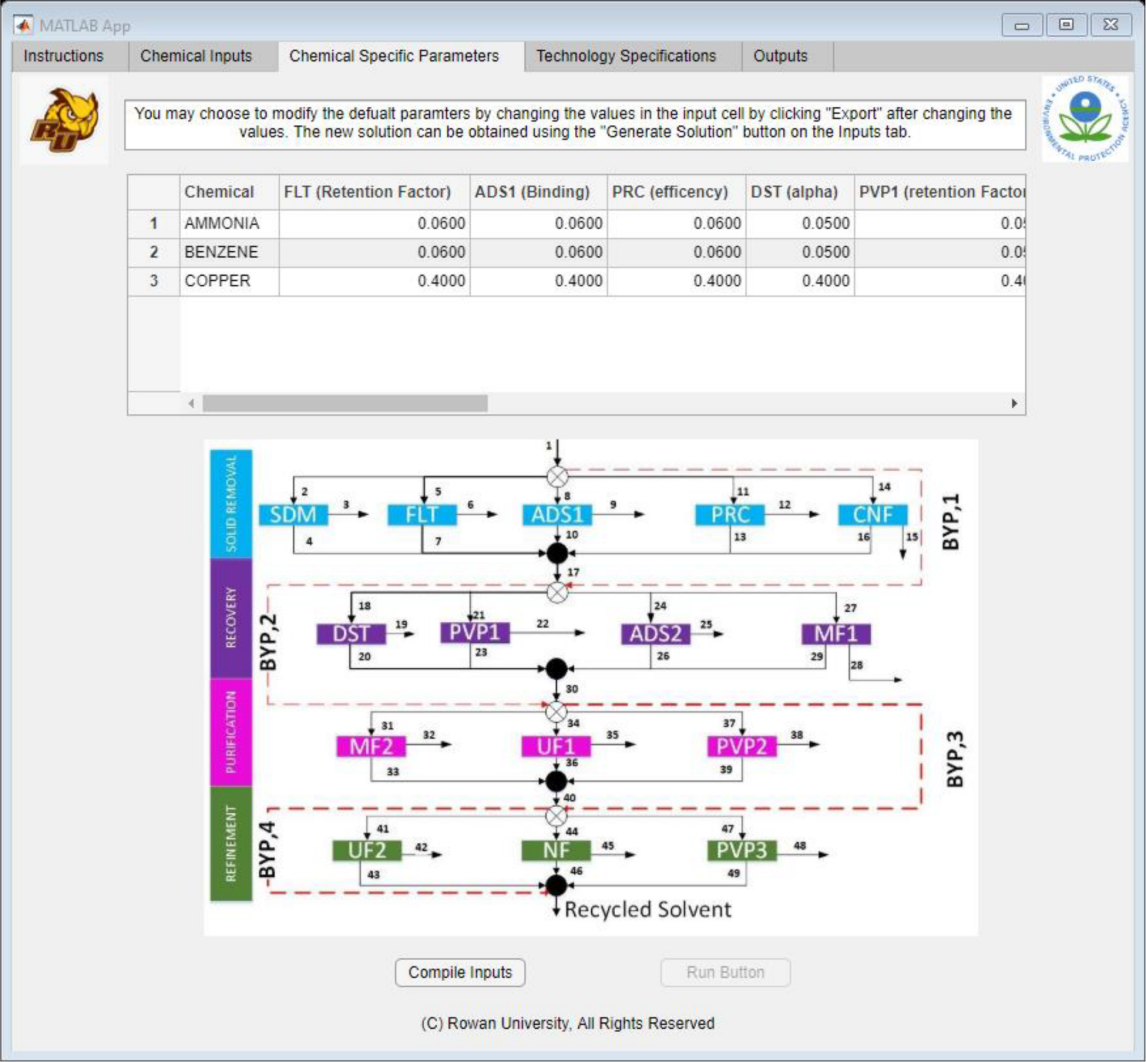
Application interface built using MATLAB App Designer, showing the “Chemical Specific Parameters” tab.

After the “Continue” button was selected, the application simultaneously saved the inputs and moved onto the second tab: “Chemical Specific Parameters”. This tab focused on technology specifications that varied with each chemical present in the waste stream. All the parameters that varied with each chemical were combined in a table found at the top of Figure 7. This table was automatically populated with estimated values based on the previous inputs from the tab in Figure 6. In addition, the user could change any of the values for any technology and component in the table in Figure 7. Once these inputs were to the user’s liking, the user would select the “Compile Inputs” button, which would save all the inputs in Figures 6 and 7, and all the saved information was sent to Excel. After the information was compiled in Excel, the “Run Button” would activate, allowing the user to run the algorithm. At this time, the “Run Button” will use the options that the team selected for options, such as the main solver, maximum execution time, and the integer optimality gap. This was decided by the team to reduce the amount of licenses needed by the user and make this tool available at the lowest possible cost to any user.

By clicking on the “Run Button” the GAMS code would get a command from MATLAB to execute the algorithm with the new inputs and the default values from the “Technology Specifications” tab. If the user wanted to change these default settings and values,the user would have to navigate to the “Technology Specifications” tab.

After the user navigates to the “Technology Specifications” tab, they will see the default values for all technologies in the superstructure. As shown in Figure 7, the user could use the drop-down menu to select any technologies in the superstructure, and the table on the right would populate with all the parameter values.

**Figure 8:**
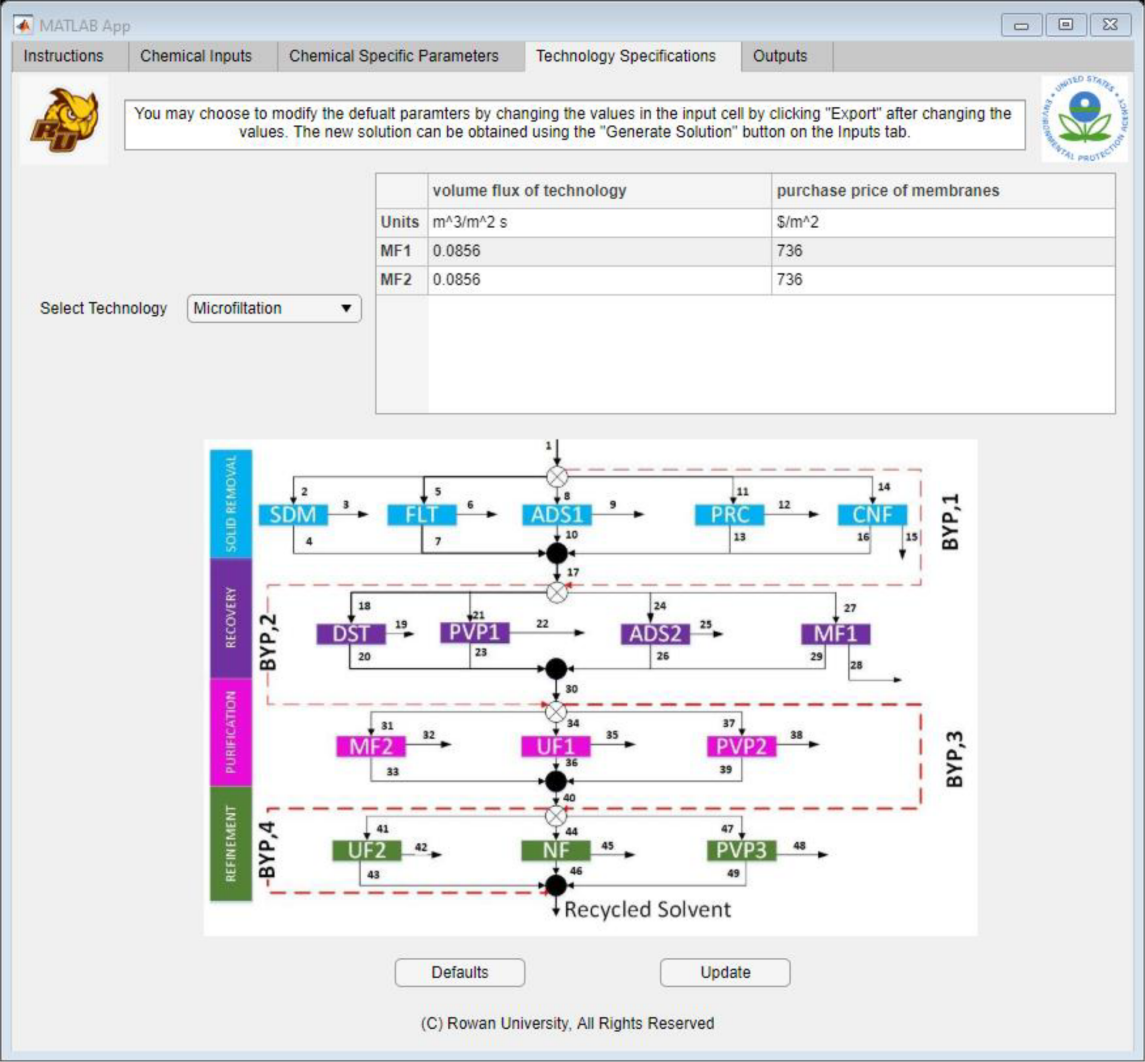
Application interface built using MATLAB App Designer, showing the technology specifications.

All the values seen in Figure 8 were variables that were in the technology model equations. A sample set of equations for the membrane technology models^31,36,39,40^ is given in eqs 1 and 2.

Retention factor (*ξ*_*k,i*_) equation for the membrane:
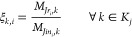
1

Area (*Qc*_*i*_) equation for the membrane:
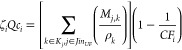
2Both equations are comprised of parameters and variables. The parameters were values that were taken from the user “Chemical Inputs” tab or the “Technology Specifications” tab, while the variables were calculated from other equations. For both equations, the parameters were ξ_*k*,*UF*_, *M*_*j*,*k*_, ζ_*UF*_, and ρ_*k*_. All these values needed to be given for the model to calculate and generate an answer. Some of these values were given by the user in the “Chemical Inputs” tab, such as *M*_*j*,*k*_, and ρ_*k*_. Other parameters such as ξ_*k*,*UF*_ were given by the “Chemical Specific Parameters” tab. The rest of the parameters were assigned default values (refer to the Supporting Information) to allow the user to get a simple economic analysis. By using the “Technology Specifications” tab, the user could modify these values to their liking and get answers that represented their system better. Once all the values were modified, the user could use the “Update” button to change all the values in the Excel sheet, which, in turn, would modify the inputs sent to the GAMS code. However, if the user wanted a simple answer, this tab could be bypassed completely, and the user would not need to interact with this tab.

**Figure 9:**
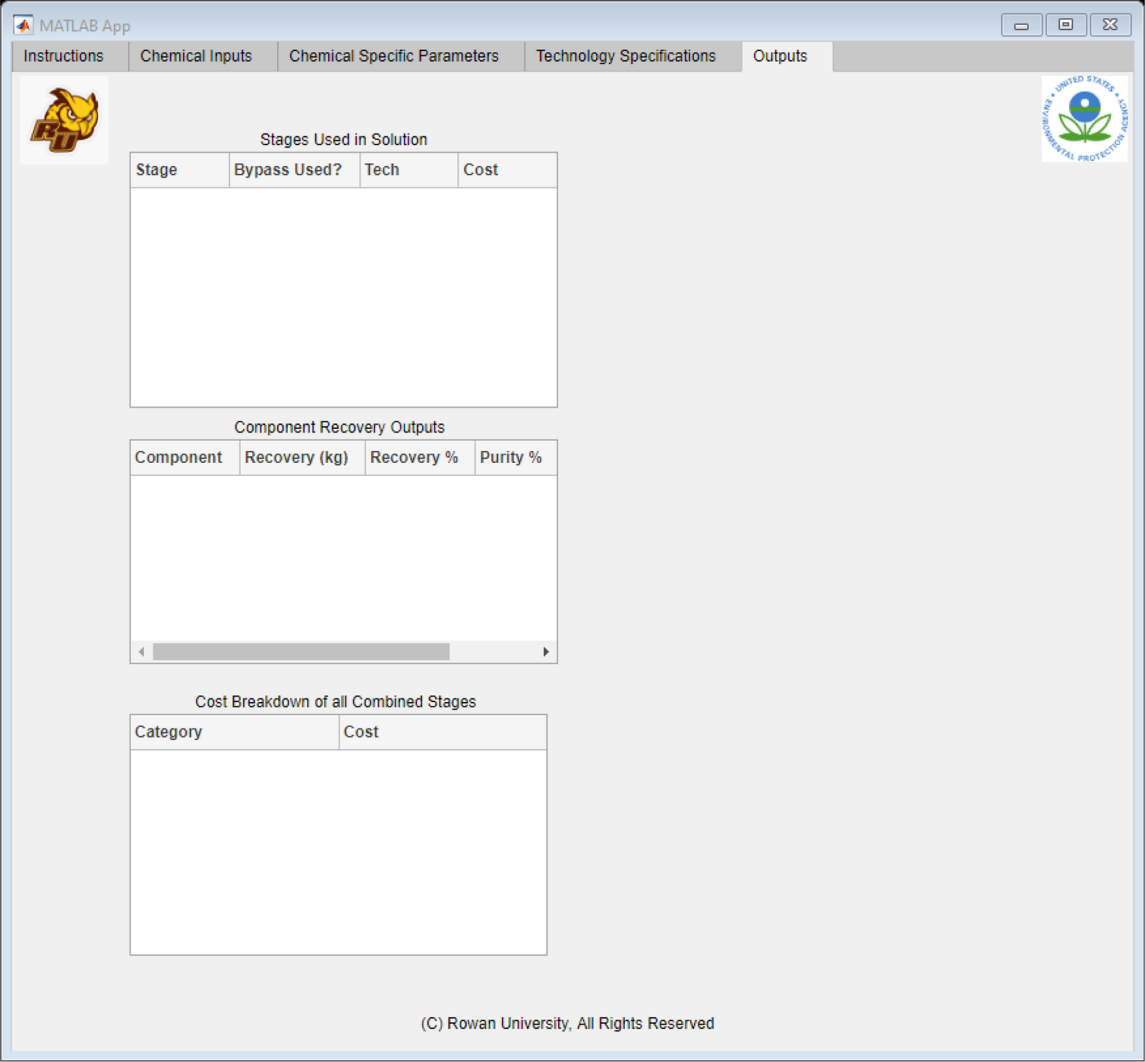
Application interface built using MATLAB App Designer, showing the “Outputs” Tab.

Figure 9 shows the general structure of the expected outputs. After selecting the “Run Button”, the application would signal the algorithm to run with the new inputs. After the code has completed running, the results would be compiled and sent back to Excel. MATLAB then reads these Excel spreadsheets, interprets them, and allocates the results to the corresponding tables in the tab, as shown in Figure 9. The “Outputs” tab generated three different reports: stage, component, and cost breakdown. The stage report displayed the optimal technology selections at each stage and reported a stage cost breakdown. This comparison allowed the user to see what stage or technology takes precedent in the solvent recovery method. Alternatively, if the user had a spare technology similar to the suggested optimal solution, this breakdown showed different methods in which the technology can be used. The component breakdown gave an analysis of the purified waste stream. The analysis showed the purity of each component in the purified stream, along with the recovered mass. With this information, the user could calculate the amount of solvents that can be recycled in the process, which would minimize the waste and the need to purchase large quantities of new solvent. Using these figures, the user could calculate how much the facility was saving on raw material costs and compare it to the total cost breakdown of the next report. The total cost was categorized into six categories: capital, labor, utilities, consumables, overhead, and materials. By comparing the total cost to the savings from the recovered materials, the user could form an economic assessment of the optimal pathway and determine whether to proceed with more-detailed process modeling.

## 3 Results and Discussions

The case studies used in this section are used to demonstrate the capabilities of the algorithm to handle inputs from multiple industries. Background for each case study is introduced, the model inputs are shown, and outputs from the tool are displayed. The first case study is a binary mixture of water and isopropanol from the pharmaceutical industry. The second case study is a four-component mixture from the recycling industry. These results match those from previous work done by Chea et al.^15^

### 3.2 Case Study A: IPA/WTR

#### 3.2.1 Process Background

**Figure 10:**
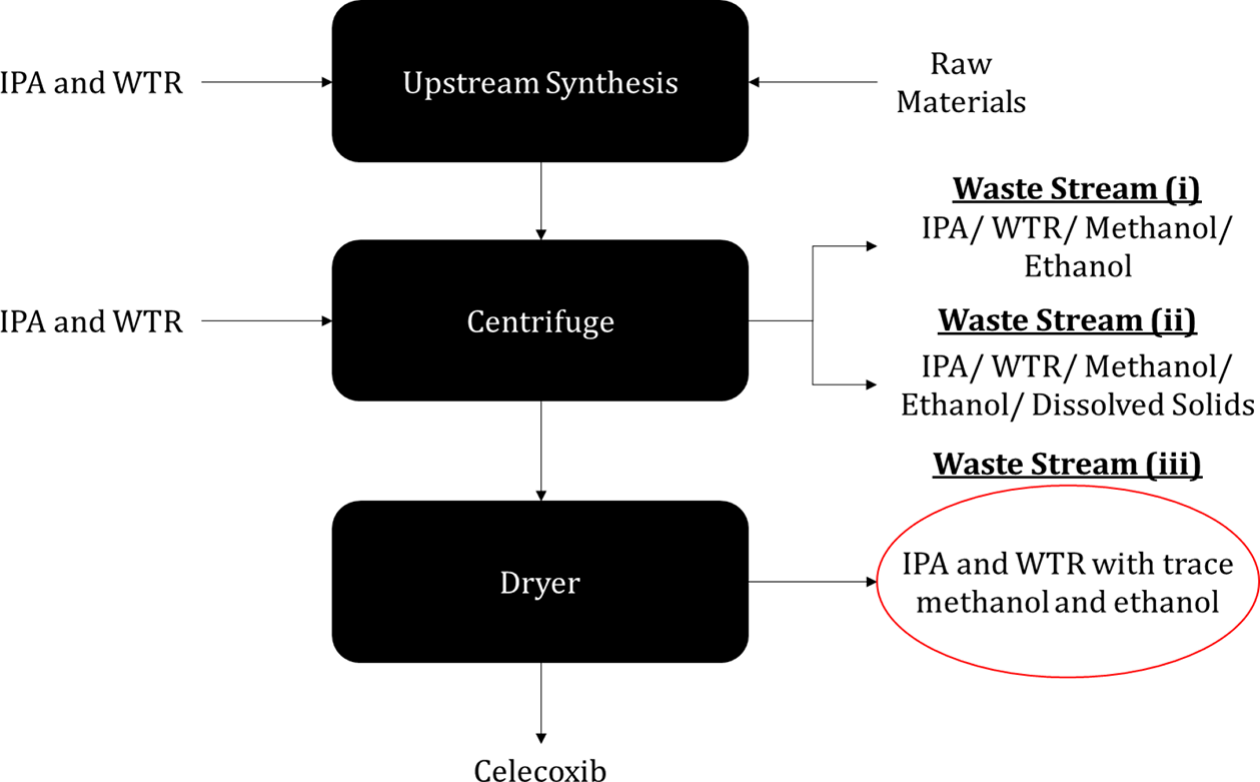
Celecoxib synthesis process showing the possible waste streams to use for solvent recovery.

This case study examined a celecoxib manufacturing process in which a large quantity of isopropanol, methanol, and ethanol waste was produced. As shown in Figure 10, following the upstream synthesis of celecoxib, the liquid product is fed through a centrifuge and dryer to obtain the purified drug.

In this process, three different waste streams were generated: (i) IPA/water washes, (ii) mother liquor (filtrate), and (iii) dryer distillates. For this case study, waste stream (iii) was used as the basis for the inputs into the solvent recovery tool. While this pharmaceutical process had a relatively low waste generation, a life cycle analysis (LCA)^3^ determined an estimated 2.19 kg total emissions/kg IPA used. Therefore, we could use the tool to find a valid solvent recovery option and reduce these total emissions.

#### 3.2.2 Waste Input Conditions

**Figure 11:**
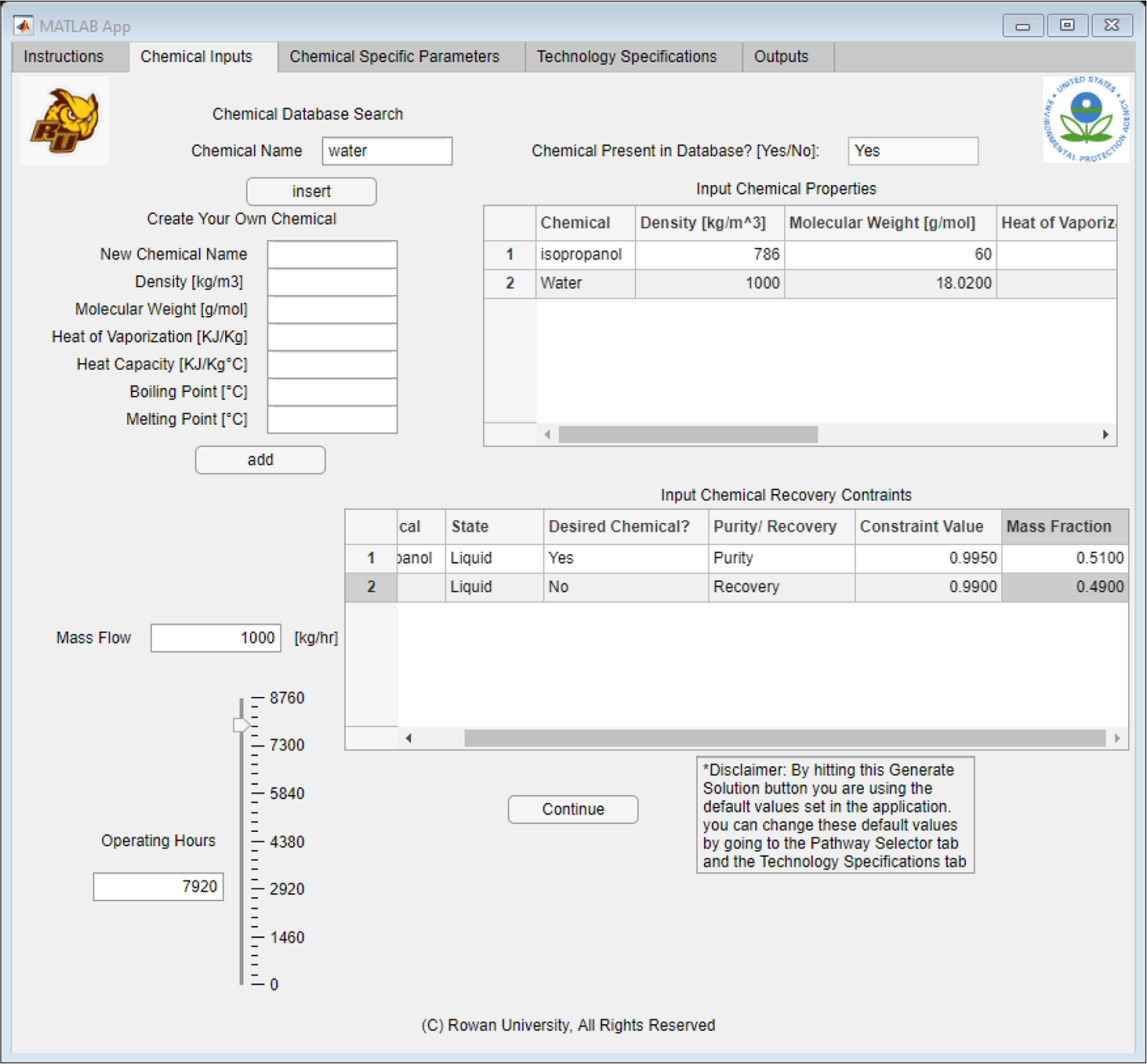
Chemical Inputs tab for IPA Recovery Case Study.

The solvent recovery tool further requires that the waste stream is fully defined. Therefore, trace components are excluded for this simple case study, leaving a binary system of isopropanol (IPA) and water. This binary system comprised 51% IPA and 49% water, with an assumed mass flow rate of 1000 kg/h. To meet stringent purity standards of the pharmaceutical industry, we aimed to achieve a purity of at least 99.5% for IPA. In addition, we aimed to recover at least 99.0% of the water. These specifications are summarized in Table 2 and inputted into the software tool, as seen in Figure 11.

**Table 2:** Waste Input Specifications for Example Case Study on IPA Recovery

component	mass (kg/h)	inlet mass fraction (%)	output requirements
isopropanol (IPA)	510	51	purity: >99.5%
water (WTR)	490	49	recovery: >99.0%

#### 3.2.3 Solvent Recovery Tool Results

**Figure 12:**
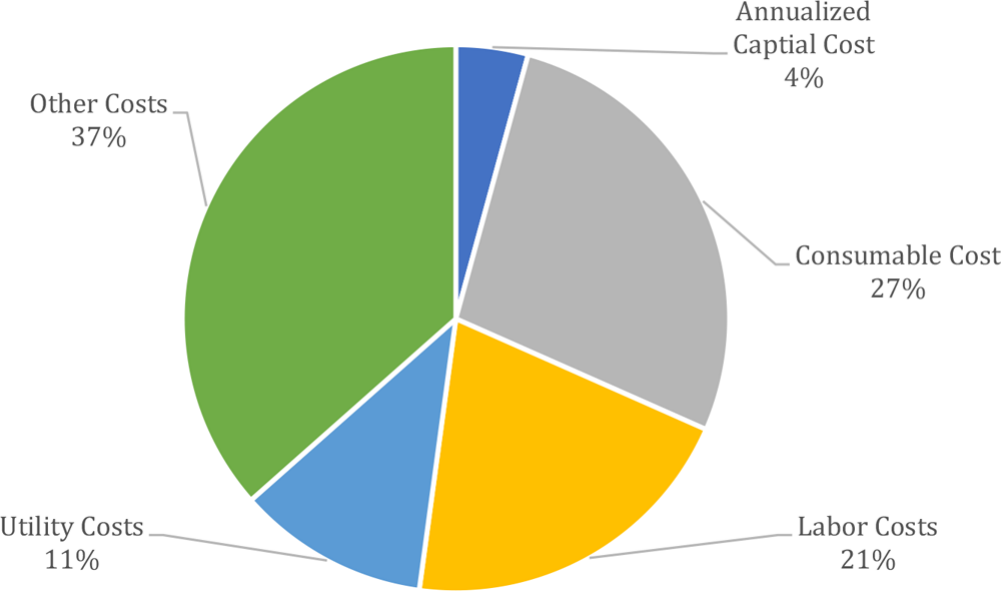
Annualized cost breakdown into economic categories for IPA Recovery Case Study, total cost = $323 000 USD.

**Figure 13:**
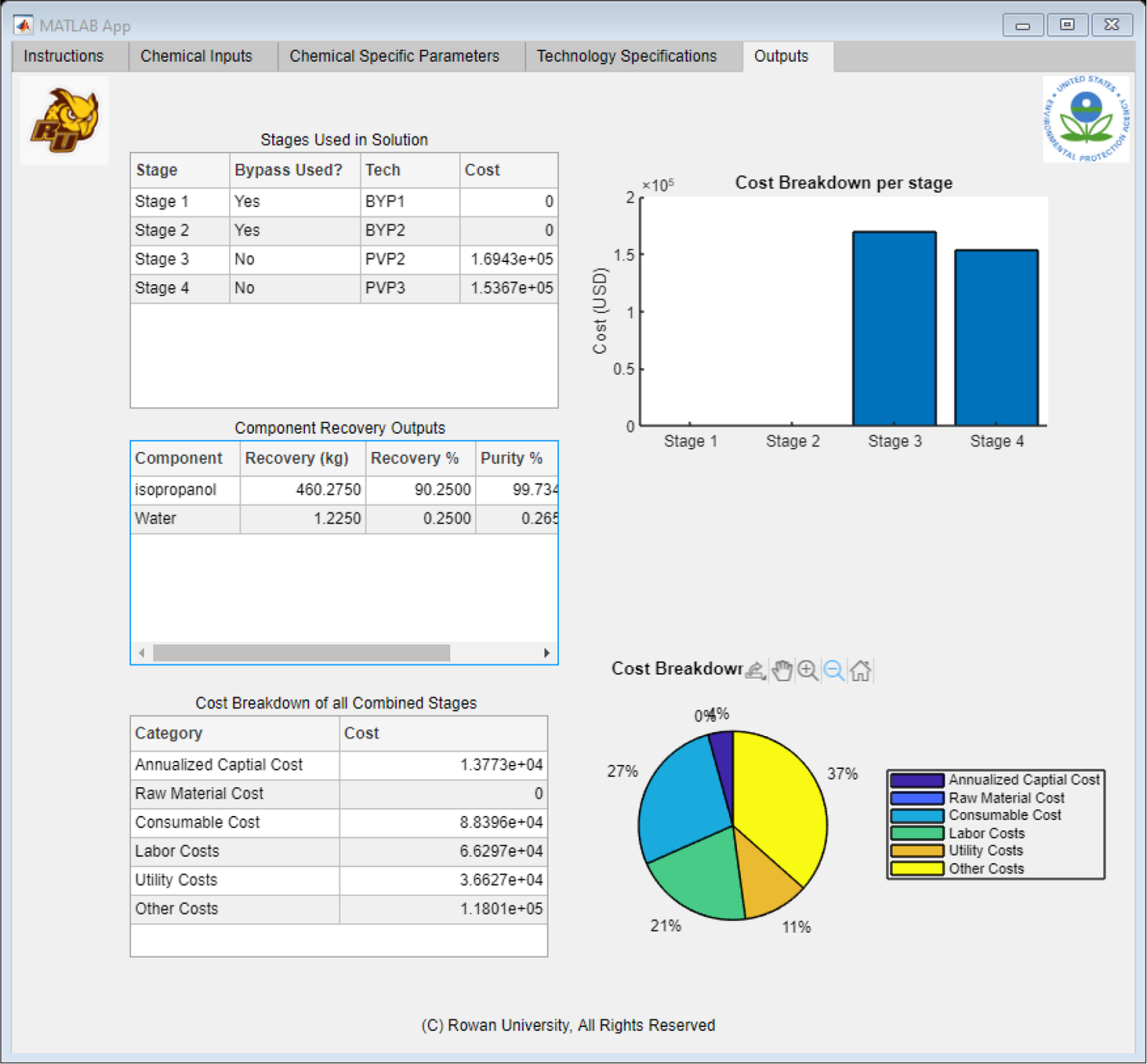
“Outputs” tab showing the completed results for the IPA Recovery Case Study.

Figure 12 and Table 3 summarize the results of the IPA recovery case study. The optimized solvent recovery pathway was BYP1-BYP2-PVP2-PVP3, which amounted to a total cost of $323 000 per year. When this waste stream was modeled in an incineration process, the cost of incineration was estimated to be $8.1 million per year. The total estimated savings for the user of this process would be $7.7 million if solvent recovery was implemented. All of this information is summarized in the “Outputs” tab of the application. This view can be seen in Figure 13.

**Table 3:** Output Mass Flow Rate of Optimized Solvent Recovery Process for IPA Recovery Case Study

	recovered solvent stream			disposable stream		
chemical	output (kg/h)	purity (%)	recovery (%)	output (kg/h)	purity (%)	recovery (%)
isopropanol	460.28	99.7	90.3	49.73	9	9.7
water	1.23	0.3	0.3	488.78	91	99.7

### 3.3 Case Study B: Ethyl Benzoate Recovery from Polyethylene Terephthalate

#### 3.3.1 Process Background

**Figure 14:**
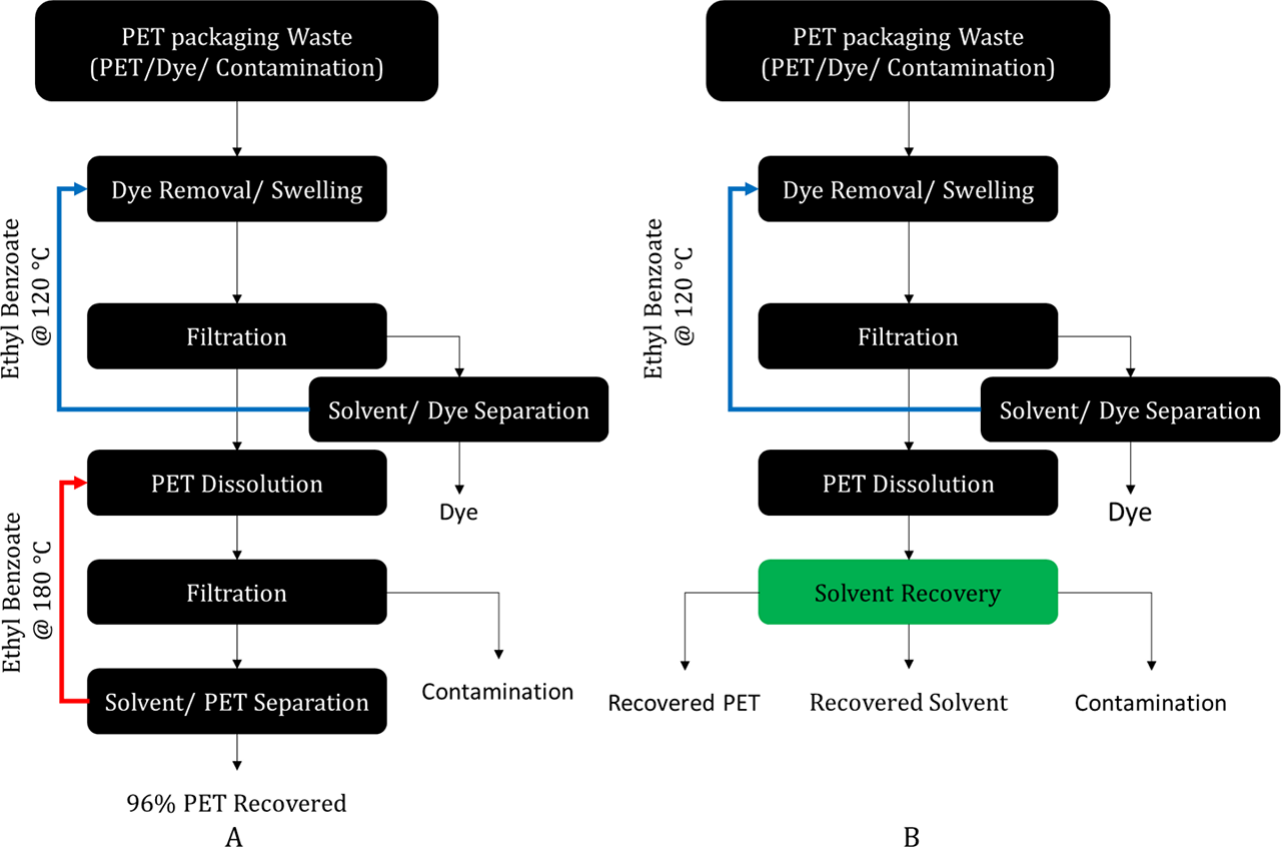
Closed-loop recycling process of post-consumer polyethylene terephthalate (PET) waste using ethyl benzoate (process adapted from ref (41)). (a) the Patented PET Recycle Approach, (b) Proposed Solvent Recovery Approach.

The second case study was derived from a recycling process of a thermoplastic polymer, and polyethylene terephthalate (PET). Figure 14A depicts the patented two-stage closed-loop recycling process that uses organic solvents to recycle post-consumer PET waste.^41^ The proposed process consisted of two key steps: (1) dye removal and (2) polymer recovery. PET waste was first subjected to a dye removal by dissolution through a solvent such as ethyl benzoate (EB) at 120 °C. The solvent at this temperature could swell the polymer and dissolve dye traces. The second step of the process used EB at 180 °C to dissolve the swelled PET fully. Any material remaining in the solid phase was removed as a contamination in the filtration step. Both steps used significant amounts of EB to complete the recycling process, which required a ratio of 22.78 g of EB: 1 g of PET. Because of the large amount of solvent used throughout the process, our solvent recovery tool could be used to find a better method to recover the solvent and polymer. Figure 14B shows the proposed changes by using the solvent recovery tool. The goal is to find another set of technologies that can purify the solvent and recover the PET while reducing the cost of the patented process.

#### 3.3.2 Waste Input Conditions

**Figure 15:**
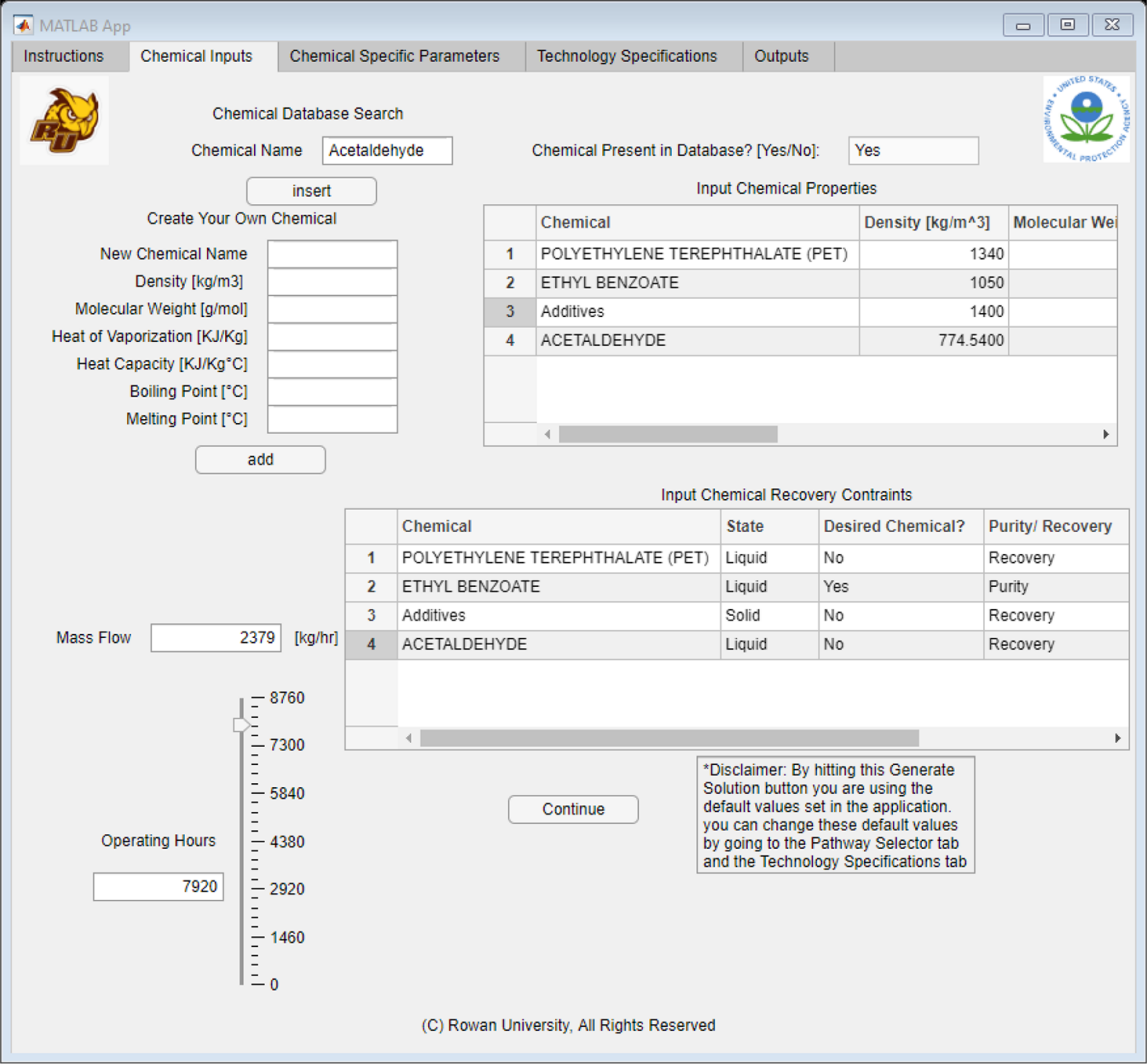
“Chemical Inputs” tab for EB Recovery Case Study.

Using the solvent–polymer ratio from the patented process, we constructed the inputs for the solvent recovery tool by setting the waste feed of PET to 100 kg/h and EB waste feed to 2278 kg/h. In addition to the EB and PET, there are trace amounts of other chemicals such as polymer additives and acetaldehyde in the mixture. The trace components are not neglected for this case study, to demonstrate the capability of the tool to handle additional complexity. In this case study, we set the waste feed for both components to 0.5 kg/h. In addition to the flow rates, we aimed to purify the solvent and recover a minimum of 96% of the polymer while removing the trace impurities. All of these specifications used in this study are summarized in Table 4. The completed tab for case study B can be seen in Figure 15.

**Table 4:** Waste Input Specifications for Example Case Study on EB Recovery

component	mass (kg/h)	inlet mass fraction (%)	output requirements
polyethylene terephthalate, PET	100	4.20	recovery: >96%
ethyl benzoate, EB	2278	95.75	purity: >99%
additives, ADD	0.5	0.02	removal: >95%
acetaldehyde, ACT	0.5	0.02	removal: >95%

#### 3.3.3 Solvent Recovery Tool Results

**Figure 16:**
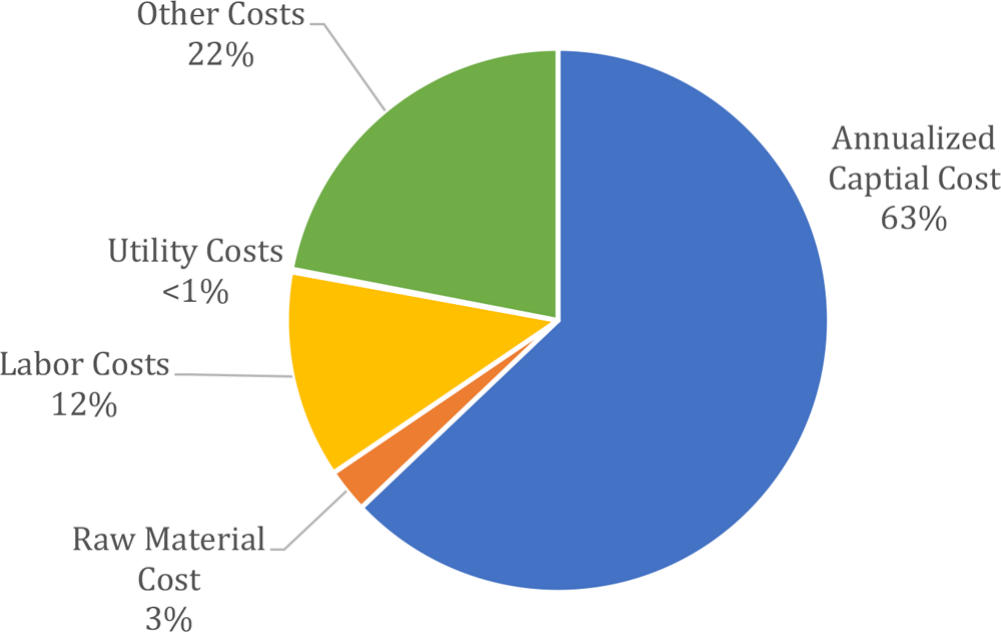
Annualized cost breakdown into economic categories for IPA Recovery Case Study, total cost = $108 000 USD.

**Figure 17:**
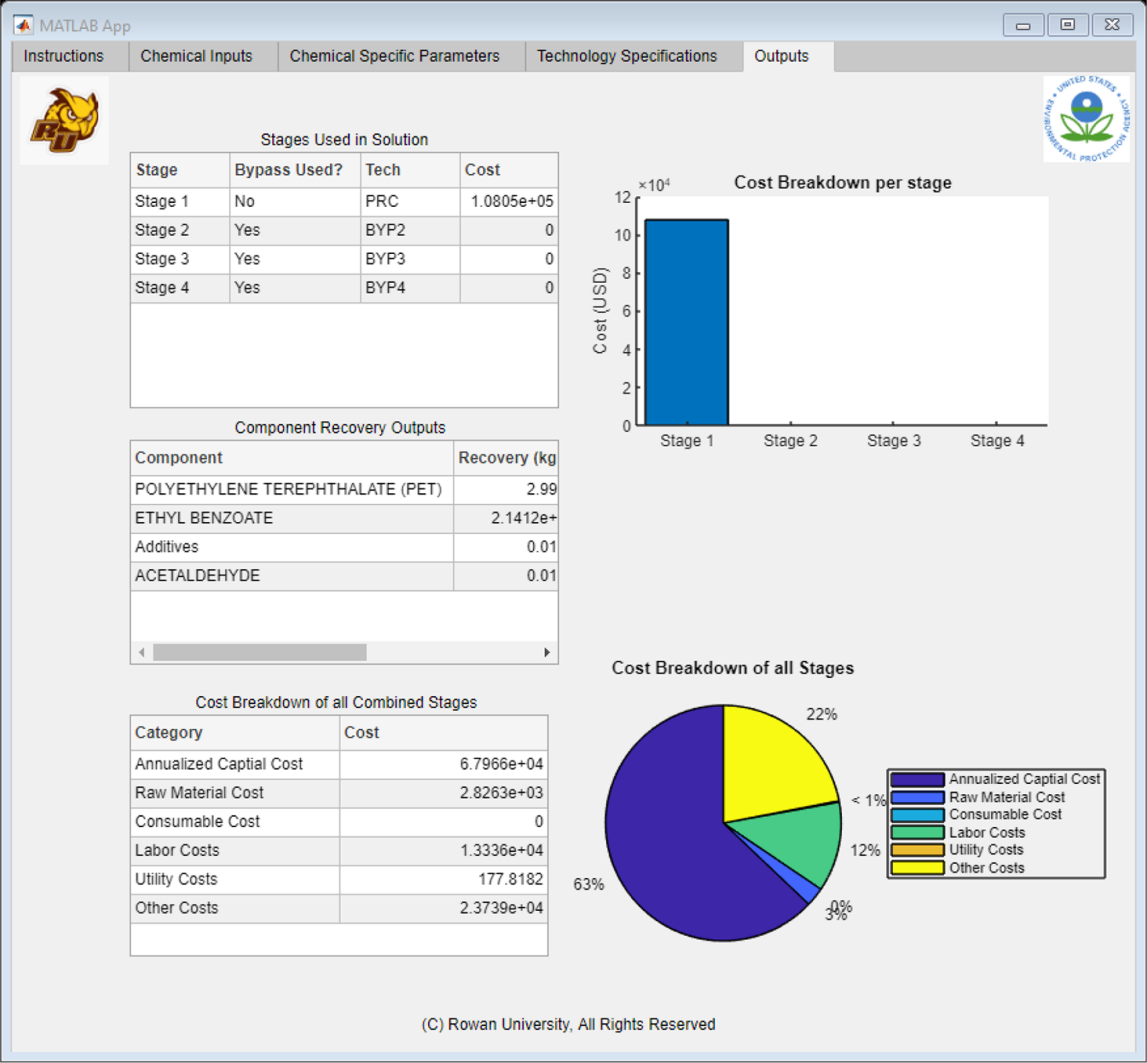
“Outputs” Tab showing the completed results for the EB Recovery Case Study.

The results of the EB recovery case study were encapsulated in Figure 16 and Table 5. The algorithm selected the optimal solvent recovery pathway as PRC-BYP2-BYP3-BYP4 and estimated the cost to be a total of $108 000 per year. When the waste stream was modeled in an incinerator, the total cost was projected to be $7.72 million per year. Therefore, the expected total savings for the user would amount to $7.6 million if solvent recovery was applied. All of this information is reviewed in the “Outputs” tab of the application. This view can be found in Figure 17.

**Table 5:** Output Mass Flow Rate of Optimized Solvent Recovery Process for PET Recovery Case Study

	recovered solvent stream			disposable stream		
chemical	output (kg/h)	purity (%)	recovery (%)	output (kg/h)	purity (%)	recovery (%)
PET	3.00	0.1	3	96.92	41	97
EB	2141.22	99.9	94	136.67	58	6
additives	0.02	0.0	3	0.58	0	97
ACT	0.02	0.0	3	0.58	0	97

## 4 Conclusions

We developed an easy-to-use software tool for the design of solvent recovery systems. The tool offers a graphical interface to quickly model potential solvent recovery systems. The user can easily input the data of a given waste stream, the purity and recovery desired, and run the model. The problem is solved in GAMS as an MINLP problem, and the results are displayed to the user on the GUI. In the future, we would like to incorporate a database management system instead of using Excel to store the data. This will allow us to publish the tool online and add more to the tool. Some aspects the team is looking to add to the model are environmental metrics and creating a method of compiling the full run statistics to send to the user. By incorporating environmental metrics, we can reformulate the algorithm to be a multiobjective problem to generate solutions with minimized cost and environmental impact. We hope that both industry and researchers will find this application useful in their design process.

## Supporting Information Available

Parameters and variables used in the case studies demonstrated via the software tool (PDF)
