# Supplementary material for: Development of an Interactive Software Tool for Designing Solvent Recovery Processes
Source: Ind Eng Chem Res. 2023 Jan 20;62(5):2090–103. doi: 10.1021/acs.iecr.2c02920 (PMC10035026; doi:10.1021/acs.iecr.2c02920)
Supplement: Supplementary file 1 — ie2c02920_si_001.pdf [file ie2c02920_si_001.pdf]

Supplementary Information (SI)

Development of an Interactive Software tool for Designing Solvent Recovery Processes

Jake P. Stengel, Austin L. Lehr, Emmanuel A. Aboagye, John D. Chea, and Kirti M. Yenkie*

Department of Chemical Engineering, Rowan University, 201 Mullica Hill Road, Glassboro, NJ, 08028, USA

S.1. Model/ Algorithm Development

Model Development

To begin designing a solvent recovery framework, a list of separation technologies capable of achieving various types of separations was compiled. Engineering design textbooks and research articles were used to allocate each technology into a separate kind of separation based on the fundamental driving forces(Wenzhao Wu, Yenkie, and Maravelias 2019; Yenkie, Wu, and Maravelias 2017; Yenkie et al. 2016; Biegler 1997; Green and Perry 2019; Ho and Sirkar 1992; Towler and Sinnott 2012). Information was acquired to correctly model the mass, energy, and design equations for each individual technology. The different types of separation technologies which were researched are found in Table S.1.

Table S.1: Researched Separation Technologies with Driving Forces and Important Considerations

Technology	Principle/ Driving force	Specifications and Important Conditions	Literature Sources
Physical Separation			
Precipitation (PRC)	charge solubility	antisolvent availability and requirements, temperature, pH change	(Green and Perry 2019; Mersmann and Kind 1988)
Sedimentation (SDM) or Decantation (DCT)	density gradient, settling velocity	size, density, tank depth, residence time	(Green and Perry 2019; Belter, Cussler, and Hu 1988)
centrifugation (CNF)	settling velocity centrifugal force	size, density, angular speed, the ratio of centrifugal to gravitational force and settling distance	(Green and Perry 2019; Agena, Bogle, and Cornish 1998; Ambler 1961)
High-Temperature Separation			
distillation (DST)	relative volatility	relative volatility >1.05, heat of vaporization, and energy requirements	(Green and Perry 2019; Towler and Sinnott 2012; Diwekar 2011; Górak and Sorensen 2014)
Membrane Processes			
membranes	particle/molecular size/permeability,	pore size, mol. wt. cutoff, average flux, pressure gradient, types of	(Green and Perry 2019; Ho and Sirkar 1992;

	sorption/diffusion pressure	membranes − MF, UF, NF, and RO	Lewis 1996; van Reis and Zydney 2007)
pervaporation (PVP)	sorption/diffusion, partial pressure	heat of vaporization, chemical potential gradient, pressure gradient, average flux, membrane selectivity	(Green and Perry 2019; Shao and Kumar 2011; Slater et al. 2012)
Liquid−Liquid Extraction			
liquid−liquid extraction (LLE)	selective partitioning of solutes	partition coefficient, the solubility of solutes, low solubility of the added solvent in water	(Green and Perry 2019; Seader, Henley, and Roper 2010; Towler and Sinnott 2012; Belter, Cussler, and Hu 1988; Birajdar, Padmanabhan, and Rajagopalan 2014; Kennedy and Cabral 1993)
aqueous two-phase extraction (ATPE)	partitioning of solute, bioselectivity	solubility, composition of two phases, molecular weight	(Sikdar et al. 1991; Benavides, Rito-Palomares, and Asenjo 2011; Asenjo and Andrews 2012; X. Wu et al. 2011; Johansson et al. 1998)

Due to the differences in driving forces, some technologies specialized in certain separations and performed some separations better than others. Therefore, the technologies were grouped together based on the separation tasks. These groups became the different technology stages in the computational algorithm. Figure S.1 shows some of the technology stage divisions that were developed for the algorithm.

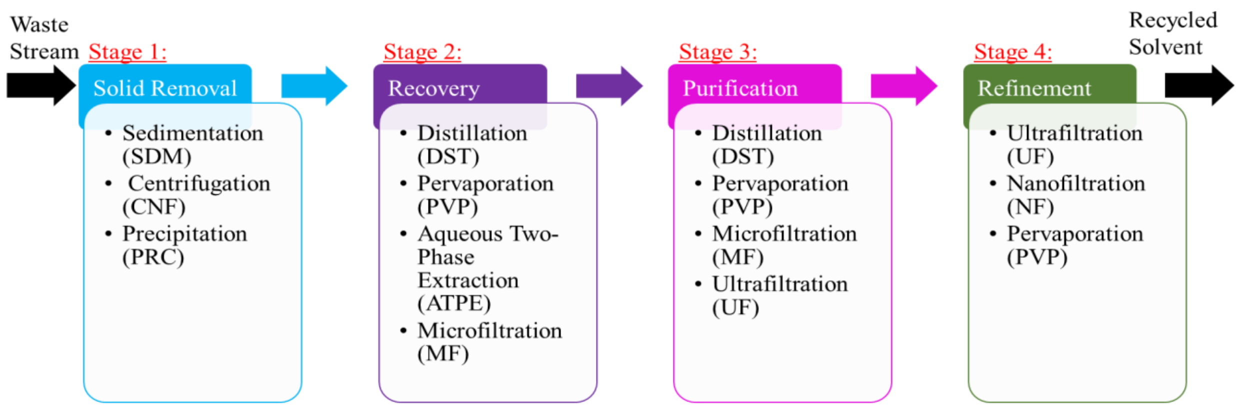

Figure S.1: Separation Stages and Technologies in the Optimization Algorithm

The four stages in the algorithm were as follows: (i) Solid Removal, (ii) Recovery, (iii) Purification, and (iv) Refinement. The solid removal stage removed any solids from the waste stream if present. The recovery stage did the major separations which retained most of the solvent. The last two stages were there to reach any purity regulations the industry needed. If any stage was not needed it could be bypassed completely, thus contributed no cost to the model. In addition, if a technology could work in multiple stages, it could be repeated multiple times. With all the stages, technology models, and chemical properties defined an optimization algorithm could be developed to synthesize pathways for the recovery of solvents from process wastes.

Algorithm Development

The optimization models consisted of three subparts: material, design, and cost analyses. The analysis showed what material was recovered from the waste stream while the design analysis displayed all parameters to correctly model the separation technology. The cost analysis provided insights into the recovery option and showed how much the new technology pathway would cost if the industry were to construct and operate it. Within this cost analysis, there were six major categories: capital, labor, utilities, consumables, overhead, and materials cost, if applicable for the specific case. The capital cost was annualized over the assumed plant life of 25 years, and a Capital Recovery Factor (CRF) of 0.11 was used to calculate the capital cost. The process was modeled as a continuous operation with a standard 330 working days a year (7920 hours). This standardized operating time allowed for the other time-dependent costs to be calculated. For labor costs, the workers' wage was estimated to be $30/hour. The utilities considered in the calculation were the

electricity, steam, and cooling water needed by the optimal solution. Any materials that were consumed and require replacement contribute to the consumable cost. Overhead costs were related to the labor costs and were any costs associated with project management. Lastly, if any raw materials were added to the process, these costs were accounted for in the materials costs. Technologies were sized using a cost-to-capacity methodology shown in equation 1.

$$\frac{C_{c_i}}{C_{0_i}} = \left(\frac{Q_{c_i}}{Q_{0_i}}\right)^n \tag{1}$$

Due to the complexity of designing a solvent recovery process, a superstructure approach was taken. The superstructure approach simultaneously compared all options in a stage and optimized over each of the four stages. This advantage allowed the algorithm to compare all technology combinations seamlessly instead of modeling each pathway directly (Wenzhao Wu, Yenkie, and Maravelias 2019; 2017; Yenkie, Wu, and Maravelias 2017; Yenkie et al. 2016; Biegler 1997; WenZhao Wu, Henao, and Maravelias 2016). To show the complexity of the superstructure approach, Figure S.2 shows the completed solvent recovery superstructure.

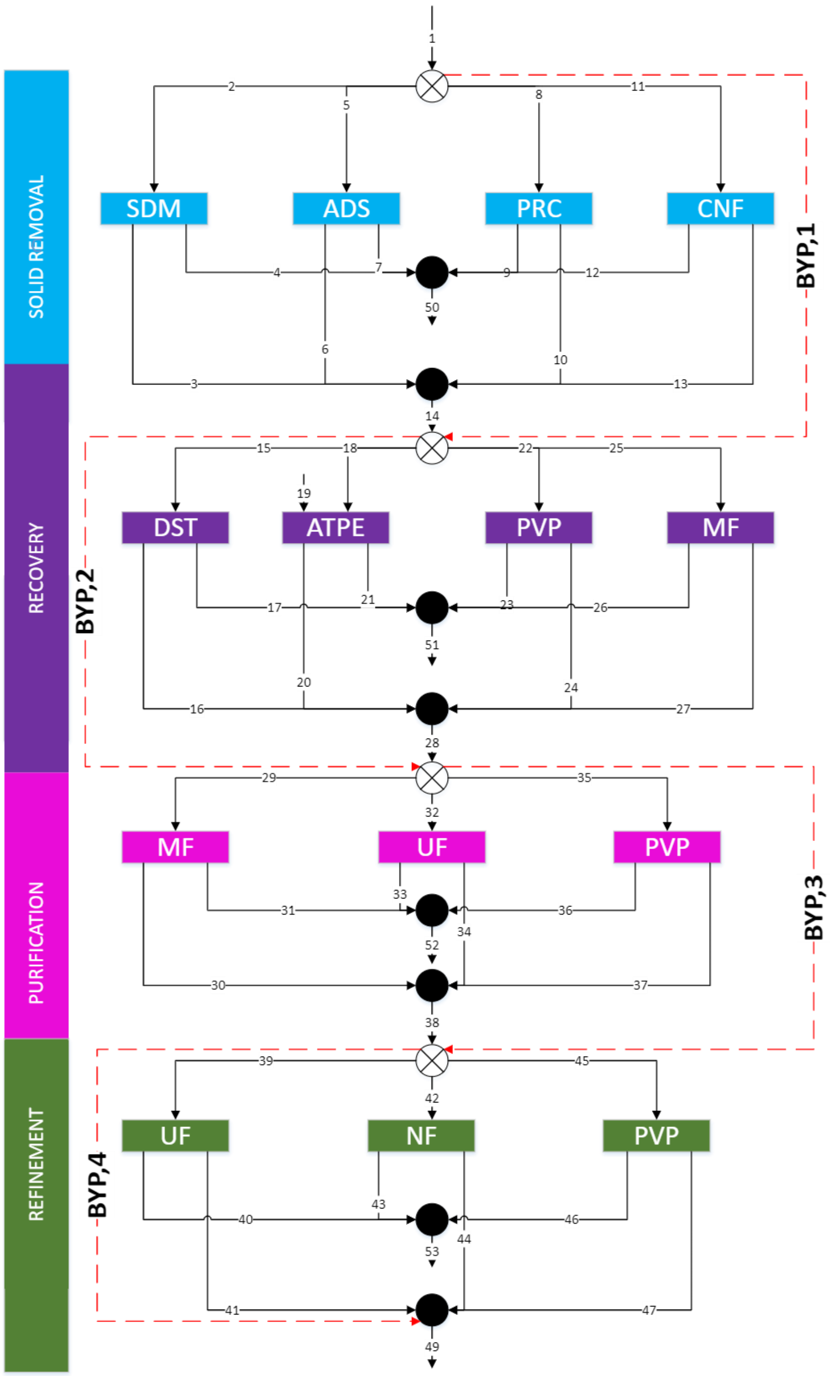

Figure S.2: Superstructure Diagram Depicting All Possible Technology Pathways
Note. SDM= Sedimentation, ADS= Adsorption, PRC= Precipitation, CNF= Centrifugation, DST= Distillation, ATPE= Aqueous Two-Phase Evaporation, PVP= Pervaporation, MF= Microfiltration, UF= Ultrafiltration, NF= Nanofiltration

Each row in Figure S.2 represented the four stages in the algorithm, while each box in the row represented a technology model. The red lines represented the bypasses, which could skip the

corresponding stage if not needed. The black circles represented mixers where purity and recovery specifications could be set for the components in the waste stream. Finally, the white circles replicated the incoming process stream so that all technologies in the stage had an identical process feed stream.

Algorithm's selective process

Each technology was modeled in GAMS as an MINLP problem with the superstructure as the starting point. MINLP was an optimization problem that employed a combination of continuous, linear, nonlinear, and binary variables to calculate the optimal solution (Biegler 1997; Diwekar 2010). Combining all the different technology models introduced continuous, linear, and nonlinear equations in the optimization problem, requiring the use of MINLP. Binary variables were assigned to each technology to ensure that only one technology per stage was selected. This was accomplished with an equation consisting of binary variables which were assigned to each stage. The equation which was assigned to each stage was seen in Equation (2)

$$\sum y_i = 1 \; \forall \; y_i \in \{0,1\}, \qquad i \in \{DST, PVP, MF, etc.\} \tag{2}$$

By utilizing this equation, only one technology would be chosen in each stage. The y in the above equation represented the binary variable, either 0 or 1. The i represented the technologies that were used in the stage for which the algorithm was solving the system of equations for optimal cost. Therefore, if the tool was optimized over stage 1, i consist of sedimentation, adsorption, precipitation, and centrifugation. This feature allowed only one technology to be chosen for each stage in the optimization algorithm, which was necessary for the superstructure approach taken in this research. The objective for this model is the minimized summation of the six cost categories previously discussed. The binary variables can select the most cost-effective recovery options based on the recovery and purity constraints of the user.

S.2. Model Equations for Technologies Used in Superstructure

List of information applicable to all technologies

S.2.1 Indices and Sets

i ∈ ***I*** – technologies (used as subscript to variables)

{ADS – Adsorption,

BYP – Bypass,

CNF – Centrifugation,

DST – Distillation,

FLT – Filtration,

INCN – Incineration,

MF – Microfiltration,

MIX – Mixer,

NF – Nanofiltration,

PVP – Pervaporation,

PRC –Precipitation,

SDM – Sedimentation,

SPL – splitter,

UF – Ultrafiltration}

$j \in \boldsymbol{J}$ – stream (used as subscript to variables)

{1, 2, 3, 4 …}

$k \in \boldsymbol{K}$ – components (used as subscript to variables)

Nstg ∈ **Nstg** – Stage numbers (used to subscript stagewise costs)

{1,2,3,4}

S.2.2 Subsets

Subsets for Technologies

I^{CST} – technologies with costs

{ADS, CNF, DST, FLT, INCN, MF, NF, PVP, PRC, SDM, UF}

I^{MEM} – technologies with membranes

{FLT, MF, NF, PVP, UF}

I^{NCST} – technologies with no cost

{BYP, MIX, SPL}

I^{CONS} – technologies with consumables

{ADS, FLT, MF, NF, PVP, UF}

I^{RMC} – technologies raw material costs

{PRC}

I^{CF} – technologies with concentration factor

{CNF, FLT, MF, NF, PVP, SDM, UF}

Subsets for Components

Jda_{DRY} – dry air inlet stream to DRY

$Jliq_{CNF}$ – stream containing no solids leaving CNF

Jin_i – inlet streams of technology *i*

$Jout_i$ – outlet streams of technology *i*

$Jsld_{CNF}$ – stream containing solids leaving CNF

Jtp_{ATPE} – top phase of ATPE

K_i – components *k* in technology *i*

K_j – components *k* in stream *j*

K^{JP} – components in process streams

S.2.3 General Parameters

ρ_k (kg/m^3) = Density of component k

π_{feed} ($/kg biomass) = Entering feed cost in terms of per kg waste

$\pi^{Rep}{}_i$ ($/unit) = Replacement cost of consumables per unit capacity in technology *i*

λ_{stm} (kJ/kg) = Latent heat of steam

$\lambda_{vap,k}$ (kJ/kg) = Heat of vaporization of component k

α_k = Relative volatility of component k for technology i

μ (N-s/m^2) = viscosity of fluid

η_{stage} = stage efficiency

θ_i^R (hr) – residence time in technology *i*

θ_i^{Rep} (h/year) = Replacement time for consumables in technology *i*

τ_{ann} (h/annum) = (330 days x 24 h/day = 7920 hours)

$C0_i$ ($/capacity) = Cost of a technology with standard capacity

Cp (KJ/kg-°C) = Specific heat of component k

$D_{p,SDM}$ = particle diameter in sedimentation unit

g (m/s^2) = gravitational constant

nc = cost scaling index (2/3 rule)

$Nlabr_i$ (#/h) = # of laborers required for technology *i* per hour

MW_k = Molecular weight of component k

$Q0_i$ (m^3 or m^2 or m^3/h) = Standard capacity of a technology for costing, labor and power required

pi = geometric constant

T_{amb} (°C) = ambient temperature

Tcw_i (°C) = Cooling water temperature in (25)

Tcw_o (°C) = Cooling water temperature out (30)

c_{pw}(KJ/Kg*C) = heat capacity of water (4.18)

T_{sat} (°C) = saturation temp

S.2.4 Evaluated Parameters

SOR_i (m/s) = surface overflow rate id sedimentation

U_i (m/s) = settling velocity of technology *i*

S.2.5 General Variables

B_i = volume ratio of equipment *i*

$Cc_{,i}$ ($) = Purchase cost of unit *i*

$\mathcal{C}F_i$ (m^3/m^3) = Concentration factor for technologies $i \in \boldsymbol{I^{CF}}$

$Cpur_k$ ($/h) = Purchase cost of added components (k ∈ $\mathbf{K^{ADD}}$)

D_i (m) = diameter of technology unit *i*

L_i (m) = length of technology unit *i*

Liq_{DST} = liquid molar flowrate in distillation column

$M_{j,k}$ (kg/h) = Mass flowrate of component k in stream j

N = actual number of stages

N_{min} = minimum number of stages

q = quality of mixture (for distillation, entering feed quality)

$\mathcal{Q}c$ (m^3 or m^2 or m^3/h) = Costing variable for technologies $i \in \boldsymbol{I^{CST}}$

QC_{DST} = cooling requirement for distillation unit

QH_{DST} = heat duty for distillation unit

Qs_{DST} = heat required to bring the feed to saturation

$\mathcal{P}W_i$ (kW/h) = Power required for technologies $i \in \boldsymbol{I}^{CST}$

R_{min} = minimum reflux ratio

R = actual reflux ratio

U_v = Underwood variable

Vap_{DST} = vapor molar flowrate in distillation column

Wsp_i(kW/h) = Power required by technology *i* per hour

$X_{mj,k}$ = mole fraction of component *k* in stream *j*

Notes:

- The 'uppercase italic Latin fonts (not colored)' are for variables (values determined through the solution of the optimization problem)
- The uppercase Latin font and lowercase Greek fonts in red are the specified input parameters
- The parameter or variable to be evaluated is always on the L.H.S of the equation

S.2.6 Evaluated Costing Variables

Cc_{Nstg} ($)– Purchase cost of each technology in stage *Nstg*

$Nlbr_{Nstg}$– Number of laborers of each technology in stage *Nstg*

PW_{Nstg} ($/hr) – Power requirement of each technology in stage *Nstg*

$Mstm_{Nstg}$ ($/hr) – Mass of steam requirement of each technology in stage *Nstg*

CRM_{Nstg} ($/hr) – Raw material cost of each technology in stage *Nstg*

CSC_{Nstg} ($/hr) – Consumable costs of each technology in stage *Nstg*

int = interest rate for Capital Recovery Factor, estimated to be 10%

PL = plant life expectancy, value is estimated to be 25 years

S.2.7 General Equations

Component balances:

$$\sum_{j \in jin_i} M_{j,k} = \sum_{j \in jout_i} M_{j,k}$$

Cost of technology i (Q_{ci}):

$$\left(\frac{C_{ci}}{C0_i}\right) = \left(\frac{Q_{ci}}{Q0_i}\right)^{nc}$$

Labor requirements of technology i (Nlb_i):

$$Nlb_i Q0_i = Nlabr_i Qc_i$$

Capital Recovery Factor (CRF):

$$CRF = \frac{int(1 + int)^{PL}}{(1 + int)^{PL} - 1} = \frac{0.1(1 + 0.1)^{25}}{(1 + 0.1)^{25} - 1} \approx 0.11$$

Annualized Capital Cost ($CCAC$):

$$CCAC = \frac{(1.66 * CRF * BMC * \sum_{Nstg} Cc_{Nstg})}{10^6}$$

Labor Cost ($CCLB$):

$$CCLB = \frac{(C_{lbr} * Tann * \sum_{Nstg} Nlbr_{Nstg})}{10^6}$$

Utility Cost ($CCUC$):

$$CCUC = \frac{\left(\left(\sum_{Nstg} PW_{Nstg} * C_{elec} + \sum_{Nstg} Mstm_{Nstg} * C_{stm}\right) * Tann\right)}{10^6}$$

Raw Material Costs ($CCRM$):

$$CCRM = \frac{\left(Tann * \sum_{Nstg} CRM_{Nstg}\right)}{10^6}$$

Consumable Cost ($CCSC$):

$$CCSC = \frac{\left(Tann * \sum_{Nstg} CSC_{Nstg}\right)}{Rep_{time} * 10^6}$$

Other Cost ($CCOC$):

$$CCOC = 2.78 * CCLB$$

Total Cost ($CCTC$):

$$CCTC = CCAC + CCUC + CCMC + CCOC + CCLB$$

S.2.8 Adsorption (ADS)

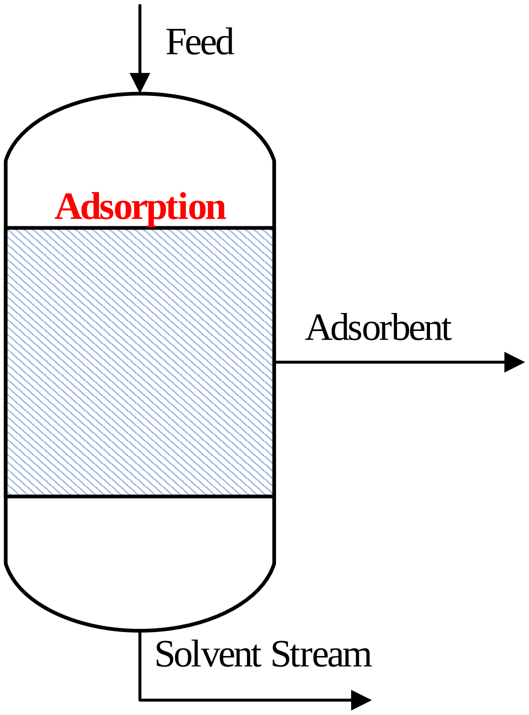

Unit Specific Parameters & Terms:

Bp_k – binding percentage of component k

EBCT (s) – empty-bed contact time

ε – void fraction

ρ_{ac} (kg/m^3) – density of activated carbon

bth (m/m)– bed to height ratio

Rep_{time} (hr) – hours of operation before absorbant media needs to be replaced

Unit Specific Model Equations:

Mass Adsorbed (m_a):

$$m_a = \sum_{k \in K_j} M_{feed,k}\, Bp_k$$

Bed Volume (V_b):

$$V_b = EBCT \sum_{k \in K_j} \frac{M_{feed,k}}{\rho_k}$$

Mass of Activated Carbon (M_{AC}):

$$M_{AC} = (1 - \varepsilon)\, V_b\, \rho_{AC}$$

Actual Capacity (Qc_{ADS}):

$$Qc_{ADS} = \frac{V_b}{bth}$$

Consumable costs:

$$CSC_{ADS} = \left(\frac{Tann}{Rep_{time}}\right) * \pi_{ADS}^{REP} * Q_{C_{ADS}}$$

Power Required (PW_{ADS}):

$$PW_{ADS} = Wsp_{ADS} Qc_{ADS}$$

S.2.9 Centrifugation (CNF)

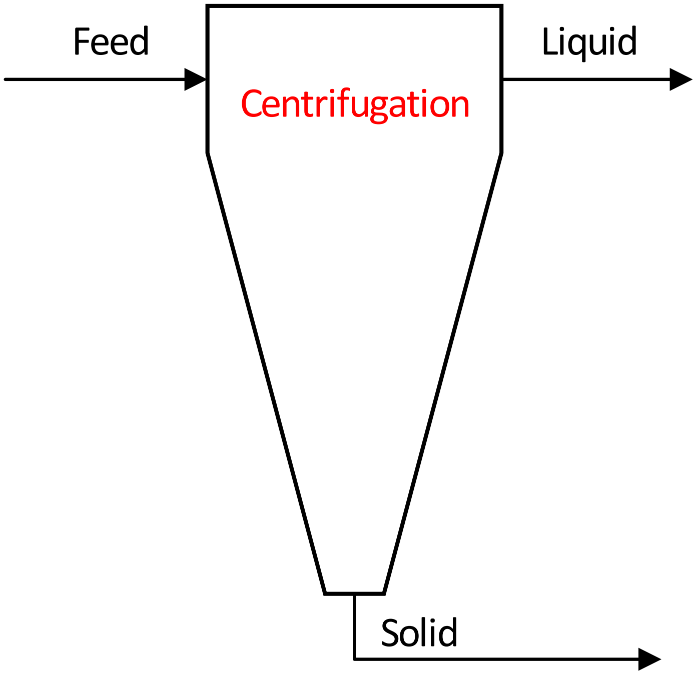

Unit Specific Parameters & Terms:

η_{water} (kg/kg) – efficiency of centrifugation on water

$\eta_{solvent}$ (kg/kg) – efficiency of centrifugation on solvent

U_{CNF} (m/hr) – sigma factor

Unit Specific Model Equations:

Efficiency Equations:

$$\eta_{water} = \frac{M_{sld_{CNF},WTR}}{M_{feed_{CNF},WTR}}$$

$$\eta_{solvent} = \frac{M_{liq_{CNF},solvent}}{M_{feed_{CNF},solvent}}$$

Concentration Factor (CF_{CNF}):

$$CF_{CNF} = \frac{\sum_{k\in K_j}\left(\frac{M_{feed,k}}{\rho_k}\right)}{\sum_{k\in K_j}\left(\frac{M_{liq,k}}{\rho_k}\right)}$$

$$2 \leq CF_{CNF} \leq 20$$

Sigma Factor Equation, Solving for Unit Size (Qc_{CNF}):

$$Qc_{CNF}U_{CNF} = \left[\sum_{k\in K_j}\left(\frac{M_{feed,k}}{\rho_k}\right)\right]$$

Power Required (PW_{CNF}):

$$PW_{CNF} = Wsp_{CNF}\left[\sum_{k\in K_j}\left(\frac{M_{feed,k}}{\rho_k}\right)\right]$$

Power Dissipation to Heat it About 40%, Therefore Cooling Duty is Required (Mcw_{CNF}):

$$Mcw_{CNF}c_{pw}(T_{CWo} - T_{CWi}) = 0.4PW$$

S.2.10 Distillation (DST)

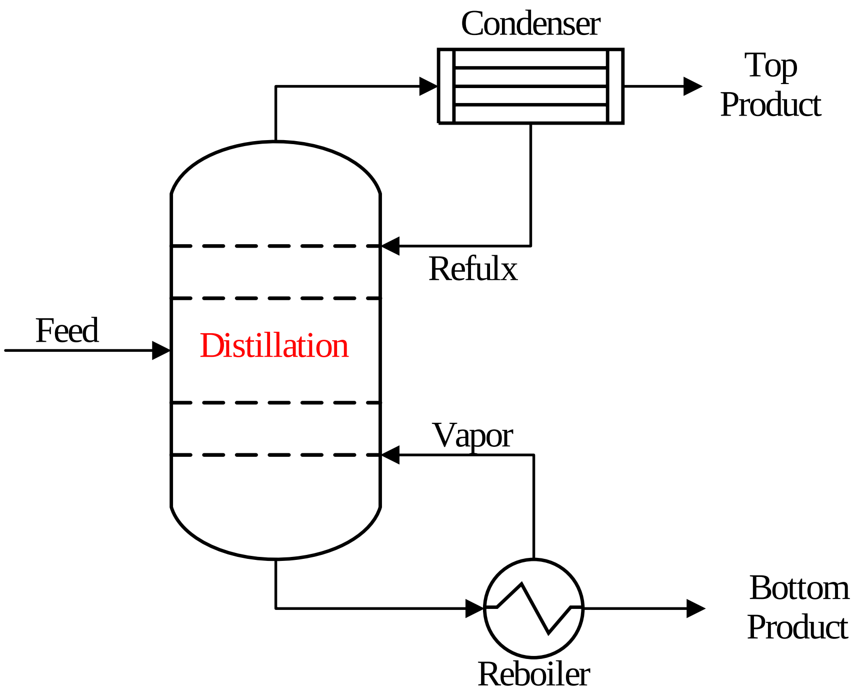

Unit Specific Parameters & Terms:

H_{stage} (m) – height of stage

HK – heavy key (Bottom Product)

LK – light key (Top Product)

Liq_{DST} – liquid molar flowrate in distillation column

η_{stage} - stage efficiency

q – quality of mixture

T- mean boiling point of the two compounds in Kelvin

t_2- boiling point in °C of heavy key

t_1- boiling point °C of light key

T_{amb} (°C) – ambient temperature

T_{sat} (°C) – saturation temperature

u_{vap} (m/s) – vapor linear velocity

Vap_{DST} – vapor molar flowrate in distillation column

$X_{mj,k}$ – mole fraction of component *k* in stream *j*

λ_{stm} (kJ/kg) – latent heat of steam

$\lambda_{vap,k}$ (kJ/kg) – heat of vaporization of component k

Unit Specific Parameter Equations:

Relative Volatility Estimation (α_k):

$$\log(\alpha_k) = \frac{t_2 - t_1}{T}(3.99 + 0.001939T)$$

Unit Specific Model Equations:

Molar Flow Rates in DST ($F_{j,k}$):

$$F_{j,k} = \frac{M_{j,k}}{MW_k}$$

Molar Component Balance in DST:

$$\sum_{j \in feed_i} F_{j,k} = \sum_{j \in out_i} F_{j,k}$$

Mole Fractions in DST:

$$X_{mj,k} = \frac{F_{j,k}}{\sum_{k \in K^{DST}} F_{j,k}}$$

Constraints on Recover, Used to Calculate Minimum Number of Stages (N_{min}):

$$X_{m_{Jtop_{DST}},k} when(\alpha_k < \alpha_{HK}) = 0$$

$$N_{min} \log(\alpha_k) = log\left[\frac{X_{m_{top_{DST}},LK}}{X_{m_{top_{DST}},HK}} * \frac{X_{m_{bot_{DST}},HK}}{X_{m_{bot_{DST}},LK}}\right]$$

Underwood's Variable (U_v):

$$(1 - q) = \sum_{k \in K^{DST}, j \in feed_{DST}} \frac{\alpha_k X_{m_{j,k}}}{\alpha_k - U_v}$$

Assume Feed is a Saturated Liquid, Making the Quality of the Mixture=0 (q=0):

$$1 = \sum_{k \in K^{DST}, j \in feed_{DST}} \frac{\alpha_k X_{m_{j,k}}}{\alpha_k - U_v}$$

Minimum Reflux Ratio (R_{min}):

$$R_{min} = \sum_{k \in K^{DST}, j \in top_{DST}} \frac{\alpha_k X_{m_{j,k}}}{\alpha_k - U_v} - 1$$

Reflux Ratio (R):

$$R = 1.3R_{min}\ (assumption)$$

Number of Stages (N):

$$0.6N = N_{min}$$

Number of Actual Stages (N_{act}):

$$N_{act} = \frac{N}{\eta_{stage}}$$

Height of Column (H_{DST}):

$$H_{DST} = H_{stage} N_{act}$$

Liquid and Vapor Flowrates (Liq_{DST}, Vap_{DST}):

$$Liq_{DST} = R \sum_{k \in K^{DST}, j \in top_{DST}} M_{j,k}$$

$$Vap_{DST} = Liq_{DST} + R \sum_{k \in K^{DST}, j \in top_{DST}} M_{j,k}$$

Column Diameter (D_{DST}):

$$D_{DST} = \sqrt{\frac{4Vap_{DST}}{\pi u_{vap}}}$$

Costing Variable of Column (Qc_{DST}):

$$Qc_{DST} = \frac{\pi}{4} D_{DST}^2 H$$

Calculating Energy Requirements of Initial Heating of Feed to Reach Saturation (ES_{DST}):

$$ES_{DST} = \sum_{k \in K^{DST}, j \in feed_{DST}} M_{j,k} Cp_k (T_{sat} - T_{amb})$$

Heat Duty Required by Reboiler (EH_{DST}):

$$EH_{DST} = (1 + R) \sum_{k \in K^{DST}, j \in top_{DST}} F_{j,k} MW_k \lambda_k^{vap}$$

Cooling Duty Required by Condenser (EC_{DST}):

$$EC_{DST} = R \sum_{k \in K^{DST}, j \in top_{DST}} F_{j,k} MW_k \lambda_k^{vap}$$

Mass of Steam Required ($Mstm_{DST}$):

$$Mstm_{DST} \lambda_{stm} = ES_{DST} + EH_{DST}$$

Mass of Cooling Water Required (Mcw_{DST}):

$$Mcw_{DST} Cp_w (Tcw_{out} - Tcw_{in}) = EC_{DST}$$

Variable Bounds:

$$N_{min} \geq y_{DST}$$

$$R_{min} \geq 1.01 y_{DST}$$

S.2.11 Membrane Models (FLT, MF, NF, UF)

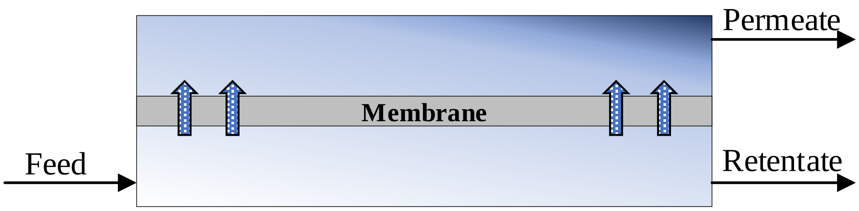

Membrane models include:

Filtration – FLT

Microfiltration – MF

Nanofiltration – NF

Ultrafiltration – UF

Unit Specific Parameters & Terms:

MEM – subset of technologies which use the filtration model (FLT, MF, UF, NF)

$\xi_{k,FLT}$ (kg/kg) – retention factor for each component k in filter MEM

ζ_{FLT} (m^3/m^2 hr) – average flux across membrane

π_{MEM}^{REP}($/unit) – replacement cost of consumables per unit capacity in membrane technologies

Rep_{time} (hr) – hours of operation before membrane needs to be replaced

Unit Specific Model Equations:

Retention Factor:

$$\xi_{k,FLT} = \frac{M_{ret_{MEM},k}}{M_{feed_{MEM},k}}$$

Concentration Factor (CF_{MEM}):

$$CF_{MEM} = \frac{\sum_{k \in K_j} \left(\frac{M_{feed,k}}{\rho_k} \right)}{\sum_{k \in K_j} \left(\frac{M_{ret,k}}{\rho_k} \right)}$$

$$lower\ bound \leq CF_{MEM} \leq upper\ bound$$

Technology	Lower bound	Upper Bound
FLT	2	30
MF	1	100
NF	0.0001	40
UF	1.01	35

Flux Balance, Solving for Unit Size ($Q_{C_{MEM}}$):

$$\zeta_{MEM} Q_{C_{MEM}} = \left[\sum_{k \in K_j} \left(\frac{M_{feed,k}}{\rho_k} \right) \right] \left(1 - \frac{1}{CF_{MEM}} \right)$$

Consumable Costs for Membrane Unit MEM (CSC_{MEM}):

$$CSC_{MEM} = \left(\frac{Tann}{Rep_{time}} \right) * \pi_{MEM}^{REP} * Q_{C_{MEM}}$$

Power Required (PW_{MEM}):

$$PW_{MEM} = Wsp_{MEM} Qc_{MEM}$$

S.2.12 Incineration (INCN)

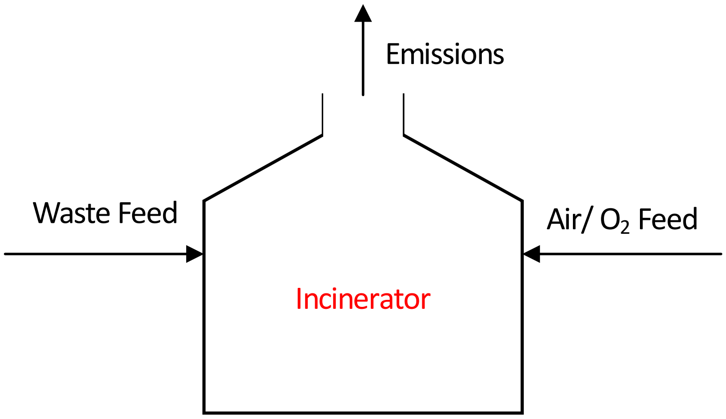

Unit Specific Parameters & Terms:

M_{waste} (kg/hr) – waste feed to the incinerator unit

C (# of carbon atoms/#atoms in mixture) – the ratio of carbon atoms to all other atoms in mixture

H (# of Hydrogen atoms/#atoms in mixture) – the ratio of Hydrogen atoms to all other atoms in mixture

O (# of Oxygen atoms/#atoms in mixture) – the ratio of Oxygen atoms to all other atoms in mixture

S (# of Sulfur atoms/#atoms in mixture) – the ratio of Sulfur atoms to all other atoms in mixture

NE_{fuel} (MJ/kg) – net energy of fuel oil = 38.9

ω (kg/kg) – ratio of air to waste feed in the incineration unit = 4.35

eff_{INCN} – Incineration Efficiency of energy production (spans between 30-40%)

C_{fuel} ($/kg) – Cost of fuel [=] $0.81/kg

$Conv_T$ (s/hr) – time conversion factor for unit consistency [=] 3600

$Conv_E$ (MJ/kWh) – energy conversion for unit consistency [=]3.6

C_{air} ($/m^3) – cost of air [=] 0.0004

Unit Specific Model Equations:

Setting the Costing Capacity:

$$Q_{ci} = M_{waste}$$

Heating Value of Waste Stream (HV_{waste}):

$$HV_{waste} = 14.544 * C + 62.208\left(H - \frac{O}{8}\right) + 4.050 * S \; [=] \; MJ/kg$$

Required Mass of Fuel Needed for Incinerating Waste (m_{fuel}):

$$M_{fuel} * NE_{fuel} = HV_{waste} * Q_{ci} \; [=] \; MJ/s$$

Air Feed Requirement Based on Waste Feed (m_{air}):

$$M_{air} = \omega * O * Q_{ci} \; [=] \; kg/hr$$

Energy Consumed During Process (E_{con}):

$$E_{con} = HV_{waste} * Q_{ci} \; [=] \; MJ/s$$

Energy Produced During Process (E_{prod}):

$$E_{prod} = eff_{INCN} * E_{con} \; [=] \; MJ/s$$

Net Energy of Process (E_{net}):

$$E_{net} = E_{prod} - E_{con} \; [=] \; MJ/s$$

Annual Fuel Consumption for a Year During Incineration (CSC_{INCN}):

$$CSC_{INCN} = M_{fuel} * C_{fuel} * Tann \; [=] \; \$/yr$$

Annual Utility Costs from Energy (E_{cost}):

$$CCUC_{INCN} = \left(\frac{E_{net} * Conv_T}{Conv_E}\right) * C_{elec} * Tann \; [=] \; \$/yr$$

Annual air cost (hydraulics and pneumatics):

$$AC_{INCN} = C_{air} * \left(\frac{M_{air}}{\rho_a}\right) * Tann \; [=] \frac{\$}{yr}$$

Capital cost:

$$C_{c,INCN} = C0_{INCN} * \left(\frac{Q_{c,INCN}}{Q0_{INCN}}\right)^{nc} \; [=] \; \$/yr$$

Number of laborers:

$$Nlb_i Q0_i = Nlabr_i Qc_i$$

$$Nlabr_{INCN} = 0.1$$

Annual cost of labor:

$$Nlbr_{INCN} = Nlb_{INCN} * C_{lbr} * Tann \; [=] \; \$/yr$$

Total annual cost (objective to be minimized):

$$CCTC_{INCN} = CSC_{INCN} + CCUC_{INCN} + AC_{INCN} + C_{c,INCN} + Nlbr_{INCN}$$

S.2.13 Precipitation (PRC)

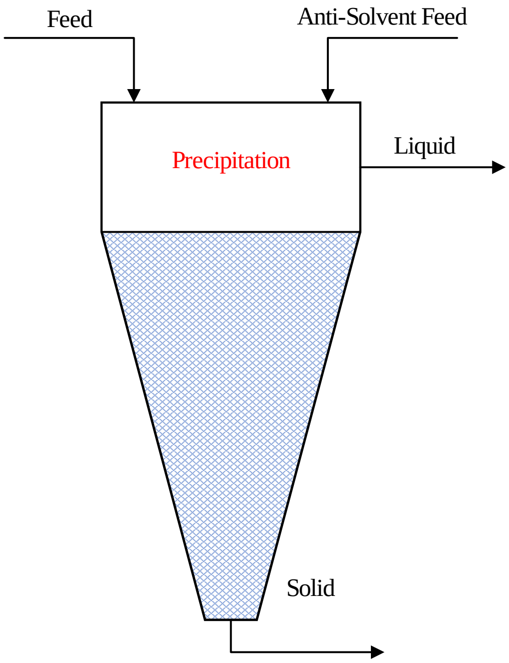

Unit Specific Parameters & Terms:

τ (h or s) – residence time

$M_{AS-feed,Ansl}$ (kg/hr) – mass of anti-solvent added for precipitation in stream j

π_{Ansl} ($/kg) – unit price of anti-solvent

φ (kg/kg) – anti-solvent to feed ratio for the addition of flocculants and enzyme

Unit Specific Model Equations:

Mass of Anti-Solvent Added for Precipitation ($M_{j,Ansl}$):

$$M_{AS-feed,Ansl} = \varphi \sum_{k \in K} M_{feed,k}$$

Consumable Costs for Precipitation Unit (CSC_{PRC}):

$$CSC_{PRC} = \pi_{PRC}^{REP} M_{AS-feed,Ansl}$$

Solving for Unit Size (Qc_{prc}):

$$Qc_{prc} = \left[\sum_{k \in Kj} \frac{M_{feed,k}}{\rho_k} \right] \tau$$

Power Required (PW_{prc}):

$$PW_{prc} = Wsp_{prc}Qc_{prc}$$

S.2.14 Pervaporation (PVP)

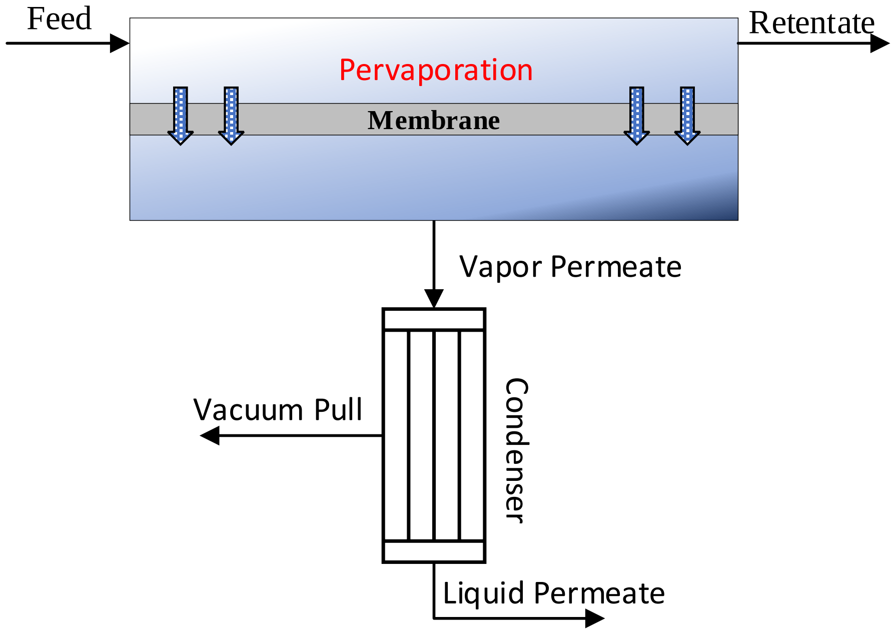

Unit Specific Parameters & Terms:

$\xi_{k,PVP}$ (kg/kg) – retention factor for each component k in PVP unit

ζ_{FLT} (m^3/m^2 hr) – average flux across membrane

λ_{stm} (KJ/kg) – latent heat of steam

$\lambda_{vap,k}$ (KJ/kg) – heat of vaporization of component k

Rep_{time} (hr) – hours of operation before membrane needs to be replaced

Unit Specific Model Equations:

Retention Factor:

$$\xi_{k,PVP} = \frac{M_{ret,k}}{M_{feed,k}}$$

Concentration Factor (CF_{PVP}):

$$CF_{PVP} = \frac{\sum_{k\in K_j}\left(\frac{M_{feed,k}}{\rho_k}\right)}{\sum_{k\in K_j}\left(\frac{M_{ret,k}}{\rho_k}\right)}$$

$$1.01 \leq CF_{PVP} \leq 35$$

Flux Balance, Solving for Unit Size ($Q_{C_{PVP}}$):

$$\zeta_{PVP} Q_{C_{PVP}} = \left[\sum_{k\in K_j}\left(\frac{M_{feed,k}}{\rho_k}\right)\right]\left(1 - \frac{1}{CF_{PVP}}\right)$$

Consumable Costs for Membrane Unit MEM (CSC_{PVP}):

$$CSC_{PVP} = \left(\frac{Tann}{Rep_{time}}\right) * \pi_{PVP}^{REP} * Q_{C_{PVP}}$$

Power Required (PW_{PVP}):

$$PW_{PVP} = Wsp_{PVP} Qc_{PVP}$$

Mass of Steam Required for Vaporization ($Mstm_{PVP}$):

$$Mstm_{PVP}\lambda_{stm} = \sum_{k\in K_j, j\in perm_{PVP}} M_{j,k}\lambda_k^{vap}$$

S.2.15 Sedimentation (SDM)

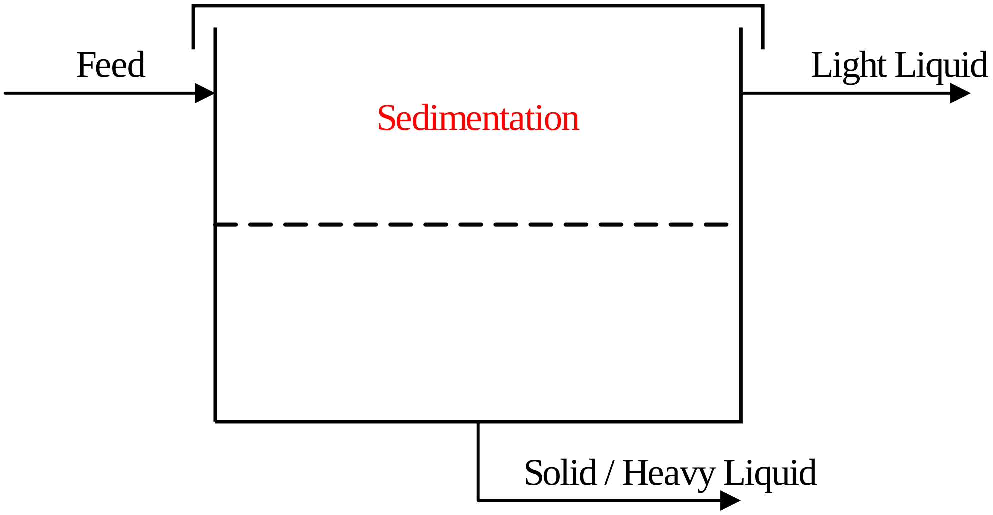

Unit Specific Parameters & Terms:

η_{SDM} (−) – efficiency of removal in typical sedimentation unit

D_p (m) – particle diameter

ρ_s (kg/m^3) – density of densest solid in mixture

ρ_l (kg/m^3) – density of liquid with highest mass fraction

μ (N-s/m^2) – viscosity of fluid

$M_{LL_{SDM},Sol}$ (kg/hr) – mass flowrate of the light liquid phase for the solvent component

$M_{HL_{SDM},Sld}$ (kg/hr) – mass flowrate of the heavy liquid phase for the solid component

Unit Specific Parameter Equations:

Settling Velocity ($U_{S,\,SDM}$) [m/s]:

$$U_{S,SDM} = \frac{gD_p^2(\rho_s - \rho_L)}{18\mu}$$

Unit Specific Model Equations:

Efficiency Equations:

$$\eta_{SDM} = \frac{M_{LL_{SDM},Sol}}{M_{feed_{SDM},Sol}}$$

$$\eta_{SDM} = \frac{M_{HL_{SDM},Sld}}{M_{feed_{SDM},Sld}}$$

Volume Concentration Factor (CF_{SDM}):

$$CF_{PVP} = \frac{\sum_{k \in K_j} \left(\frac{M_{feed,k}}{\rho_k} \right)}{\sum_{k \in K_j} \left(\frac{M_{LL,k}}{\rho_k} \right)}$$

$$1.01 \leq CF_{SDM} \leq 15$$

Surface Overflow Rate (SOR_{SDM}):

$$SOR_{SDM} = \frac{U_{S,SDM}}{\eta_{SDM}}$$

Area of Sedimentation Tank (Qc_{SDM}):

$$Qc_{SDM} = \frac{\sum_{k \in K_j j \in Jin_{SDM}} \left(\frac{M_{j,k}}{\rho_k} \right)}{SOR_{SDM}}$$

S.2.16 Degree of Freedom Analysis for Individual Technologies

Unit operation	Variables	Equations	Degrees of Freedom
Adsorption	13	12	1
Aqueous Two-Phase Extraction	18	13	5
Centrifuge	16	14	2
Distillation	14	12	2
Dryer	12	9	3
Filtration	12	11	1
Microfiltration	12	11	1
Nanofiltration	12	11	1
Ultrafiltration	12	11	1
Pervaporation	13	12	1
Precipitation	11	10	1
Sedimentation	14	11	3

S.3. Model specifications and input data for all technologies

Table S.3.1. Table for standard capacity, costs, scaling factors, labor requirements for technologies:

Technology (Costing capacity)	Standard capacity (units)	Base costs (million $)	Scaling exponent (n)	Laborers required (#/hr)	Power required (kWh)	Consumable Costs ($/unit)
ADS (volume of bed)	32 m^3	0.03	0.67	0.1	N/A	24($/kg)
CNF (Sigma factor)	60000 m^2	0.66	0.67	1	19.2	N/A
DST (Volume)	22.58 m^3	0.082	0.67	1	N/A	N/A
FLT(Area)	80 m^2	0.039	0.67	0.5	0.1	100 ($/$m^2$) [c]
MF (Area)	80 m^2	0.75	0.67	1	0.1	736 ($/$m^2$) [c]
NF (Area)	80 m^2	1.2	0.67	1	0.1	1000 ($/$m^2$) [c]
UF (Area)	80 m^2	0.938	0.67	1	0.2	874 ($/$m^2$) [c]
INCN (Mass flowrate)	100000 kg/hr	.967	0.67	0.1	~ [b]	N/A
PVP (Area)	80 m^2	0.0261	0.67	1	0.33	1000 ($/$m^2$) [c]
PRC (volumetric flowrate)	40 (m^3/hr)	0.474	0.67	1	0.1	0.3 ($/kg)
SDM (Area)	2500 m^2	1.128	0.67	0.1	N/A	N/A

a. This cost is the consumable cost associated with adding in the hexane and salt into the aqueous two-phase extraction unit. The unit cost of hexane is $2/kg, and the unit cost of sodium chloride salt is $0.6/kg

b. This value dependent on the composition of the incoming stream. Different compounds have different heat of combustions, which will cause variation in the power required.

c. The replacement time for all filter consumables in assumed to be 2000 hours.

Table S.3.2. Utility and labor costs (SuperPro Designer v8.5)

Utility	Parameter Identifier	Cost per unit ($/unit)
Electricity	C_{elec}	$0.1/kWH
Cooling Water	C_{cwtr}	$5E-5/kg
Steam	C_{stm}	$0.012/kg
Labor	C_{lbr}	$30/laborer*hr

S.4. Parameter Values for Technology Models for Example Case Study

S.4.1 Components used for Example Case Study

PET – Polyethylene Terephthalate

EB – Ethyl Benzoate

ADD - Additives

ACT - Acetaldehyde

S.4.2 Adsorption (ADS)

empty-bed contact time (EBCT) – 0.0167 hr

void fraction (ε) – 0.4 (assume spherical particles)

density of activated carbon (ρ_{ac}) - 4 kg/m^3

bed to height ratio (bth) - 0.5 (m/m)

Replacement cost of consumables per unit capacity in technology (π_i^{REP}) – *can be found in appendix S.3, Table S.3.1*

Component Dependent Parameters

Binding percentage of component k (Bp_k) - *values given in table (unitless)*

**Component**	**PET**	**EB**	**ADD**	**ACT**
ADS1	0.01	0.01	0.9	0.01
ADS2	0.01	0.8	0.9	0.9

S.4.3 Centrifugation (CNF)

efficiency of centrifugation on water (η_{water}) – 0.7 kg/kg

efficiency of centrifugation on solvent ($\eta_{solvent}$) – 0.7 kg/kg

S.4.4 Distillation (DST)

Height of stage (H_{stage}) – 1.5 m

stage efficiency (η_{stage}) – 0.8

quality of mixture (q) – 1 (assumed saturated feed)

ambient temperature (T_{amb}) – 20 °C

saturation temperature (T_{sat}) – 250 °C

Vapor linear velocity (u_{vap}) – 10,800 m/hr

Latent heat of steam (λ_{stm}) – 2115.68 kJ/kg

Component Dependent Parameters

mean boiling point of the two key compounds in Kelvin (T) – 592.85 K

boiling point °C of Light Key (t_1) – 213.4 °C

boiling point in °C of Heavy Key (t_2) - 300 °C

Heat of vaporization of component k ($\lambda_{vap,k}$) - *values given in table (kJ/kg)*

**Component**	**PET**	**EB**	**ADD**	**ACT**
DST	366.27	114	N/A	582.4

S.4.5 Membrane Models (FLT, MF, NF, UF)

Replacement cost of consumables per unit capacity in technology (π_i^{REP}) – *can be found in appendix S.3, Table S.3.1*

Average flux across membrane (ζ_{FLT}) - *values given in table (m^3/m^2 hr)*

**Technology**	**FLT**	**MF**	**NF**	**UF**
Values	0.2	0.0856	0.2	0.0856

Component Dependent Parameters

Retention factor for each component k in filter MEM ($\xi_{k,FLT}$) - *values given in table (kg/kg)*

**Component**	**PET**	**EB**	**ADD**	**ACT**
FLT	0.01	0.01	0.9	0.01
MF1	0.01	0.8	0.95	0.95
MF2	0.01	0.8	0.95	0.95
NF	0.01	0.97	0.5	0.5
UF1	0.01	0.97	0.95	0.95
UF2	0.01	0.97	0.95	0.95

S.4.6 Incineration (INCN)

waste feed to the incinerator unit (M_{waste}) – specified by user in kg/hr

net energy of fuel oil (NE_{fuel}) - 38.9 MJ/kg

ratio of air to waste feed in the incineration unit (ω) - 4.35 kg/kg

Incineration Efficiency of energy production (eff_{INCN}) - 30%

Cost of fuel (C_{fuel}) - 0.81 \$/kg

time conversion factor for unit consistency ($Conv_T$) – 3600 s/hr

energy conversion for unit consistency ($Conv_E$) - 3.6 MJ/kWh

cost of air (C_{air}) - 0.0004 \$/m^3

S.4.7 Pervaporation (PVP)

Replacement cost of consumables per unit capacity in technology (π_i^{REP}) – *can be found in appendix S3, Table S3.1*

Average flux across membrane (ζ_{FLT}) - 0.055 (m^3/m^2 hr)

Latent heat of steam (λ_{stm}) – 2115.68 kJ/kg

Component Dependent Parameters

Retention factor for each component k in filter MEM ($\xi_{k,PVP}$) - *values given in table (kg/kg)*

**Component**	**PET**	**EB**	**ADD**	**ACT**
PVP1	0.01	0.7	0.95	0.95
PVP2	0.01	0.7	0.95	0.95
PVP3	0.01	0.7	0.95	0.95

S.4.8 Precipitation (PRC)

Residence Time (τ) - 6 hr

Replacement cost of consumables per unit capacity in technology (π_i^{REP}) – *can be found in appendix S.3, Table S.3.1*

Anti-solvent to feed ratio for the addition of flocculants and enzyme (φ) – 2 (kg/kg)

S.4.9 Sedimentation (SDM)

efficiency of removal in typical sedimentation unit (η_{SDM}) – 0.70 (kg/kg)

particle diameter (D_p) – 0.005 m

Component Dependent Parameters

density of densest solid in mixture (ρ_s) - 1400 (kg/m^3)

density of liquid with highest mass fraction (ρ_l) - 1340 (kg/m^3)

viscosity of fluid (μ) – 8.9E-4 (N-s/m^2)

S.5. Link for the tool and associated files

https://github.com/kmygroup/Solvent-Recovery
